# Composition and role of the vacuolar transporter chaperone complex in polyphosphate synthesis and infectivity in *Trypanosoma cruzi*

**DOI:** 10.1128/mbio.00372-26

**Published:** 2026-05-13

**Authors:** Mayara S. Bertolini, Miguel A. Chiurillo, Roberto Docampo

**Affiliations:** 1 Department of Cellular Biology, Center for Tropical and Emerging Global Diseases, University of Georgia 200747, Athens, Georgia, USA; 2 Department of Biological Sciences, University of Cincinnati 118729, Cincinnati, Ohio, USA; University of Texas Southwestern, Dallas, Texas, USA

**Keywords:** *Trypanosoma cruzi*, inorganic polyphosphate, inositol pyrophosphate, vacuolar transporter chaperone

## Abstract

Inorganic polyphosphate (polyP) is a linear polymer composed of three to several hundred orthophosphate units linked by high-energy phosphoanhydride bonds and is found in both prokaryotes and eukaryotes. In *Trypanosoma cruzi*, the causative agent of Chagas disease, polyP plays important roles in osmoregulation and persistence within host tissues and is synthesized by a polyP polymerase known as the vacuolar transporter chaperone (VTC) complex. This complex, localized to acidocalcisomes, is composed of Vtc1 and the catalytic subunit Vtc4. Using CRISPR/Cas9-mediated genome editing, we generated *Vtc1* knockout (*Vtc1*-KO), *Vtc4* single knockout (*Vtc4*-SKO), and conditional knockout lines for both subunits (*Vtc1*-CKO and *Vtc4*-CKO). Analysis of these mutants revealed essential roles for Vtc1 and Vtc4 in parasite proliferation, differentiation, and egress from mammalian host cells. Moreover, co-immunoprecipitation and proteomic analyses identified a novel component of the complex, termed TcVtc6, which associates with Vtc1 and Vtc4, forms part of the VTC complex, and is involved in polyP synthesis in *Trypanosoma cruzi*.

**IMPORTANCE:**

Chagas disease affects millions of people across the Americas and remains a major unmet medical challenge. Here, we investigate the essentiality and molecular composition of the vacuolar transporter chaperone (VTC) complex in *Trypanosoma cruzi*, the causative agent of the disease. We identify a previously unrecognized component of this complex, which we term TcVtc6, and show that it is involved in polyphosphate synthesis. Functional analyses reveal that the VTC complex is indispensable for parasite differentiation and host cell egress, two processes critical for infectivity. Although the VTC complex is conserved in trypanosomatids, apicomplexans, fungi, and algae, it is absent from mammalian cells. This evolutionary divergence, together with the essential role of the pathway in infectious stages of *T. cruzi*, highlights the VTC complex as a promising and selective therapeutic target for the treatment of Chagas disease.

## INTRODUCTION

Chagas disease is the most prevalent parasitic disease in the American continent, affecting 6–8 million people, with 80 million people at risk (1). In the United States, where the disease is considered endemic (2), it is estimated that 300,000 individuals are infected and unknowingly expose others to infection through blood and organ donation (3). No vaccines are available against Chagas disease, and the current antiparasitic drug treatment has challenges, including partial or lack of activity in the acute or chronic stage of the disease, respectively, and unwanted side effects (4, 5).

A peculiarity of *Trypanosoma cruzi,* the protozoan parasite responsible for Chagas disease, is the accumulation of large amounts of polyphosphate (polyP). PolyP is stored in acidocalcisomes (6, 7), but also accumulates in glycosomes (8), nucleoli (8), and the outer surface (9) of *T. cruzi*, and is important for osmoregulation (7, 10, 11) and persistence in tissues (12).

The discovery of polyP roles in regulating the blood coagulation cascade (13), in bone mineralization (14), and in cancer (15) has resulted in a recent increase in polyP interest. Other striking discoveries are its role as an inorganic chaperone (16), its participation in the amyloidogenic process (17), the crosstalk between polyP and inositol phosphate signaling (18), and the ability of polyP to drive a protein post-translational modification (lysine polyphosphorylation) (19). In addition, bacterial polyP has been shown to interfere with the innate host defense to infection (20).

Synthesis and translocation of polyP into the lumen of acidocalcisomes are catalyzed by the vacuolar transporter chaperone (VTC) complex (21). The VTC complex is present in trypanosomatids (21, 22), apicomplexans (23, 24), fungi (25), and algae (26). The VTC complex of *Saccharomyces cerevisiae* has five subunits (Vtc1–Vtc5), of which Vtc4 is the catalytic subunit (27) and forms sub-complexes with Vtc1 and Vtc2 or Vtc3 (Vtc1/Vtc2/Vtc4 or Vtc1/Vtc3/Vtc4), which are localized to the vacuole membrane (28). Vtc5 does not form part of the sub-complexes but stimulates their activity (29). Structural studies of these sub-complexes in yeasts have shown that Vtc1 has three transmembrane domains, while Vtc2, Vtc3, and Vtc4 contain additional SPX (SYG1/Pho81/XPR1) and TTM (triphosphate tunnel metalloenzyme) domains (27, 30). The TTM domain of Vtc4 (TTM^Vtc4^) synthesizes polyP by transferring the γ-phosphate of cytosolic ATP onto the growing polyP chain, which is translocated into the vacuole (27). The SPX domains are receptors for cytosolic inositol pyrophosphates (also known as diphosphoinositol pyrophosphates, PP-IPs), which stimulate polyP synthesis (28). PP-IPs disrupt the interaction between the SPX domain of Vtc 2/3 and the SPX domain of Vtc4, stimulating its activity (31). The VTC complex of yeast has therefore functions of polyP polymerase, polyP translocase, and PP-IP receptor (30).

In trypanosomatids, the VTC complex is essential for their normal proliferation (21, 22). Trypanosomatids, as also occurs with algae (*Chlamydomonas* spp.) (26, 32), possess orthologs to Vtc1 and Vtc4 but no homologs to other subunits. Interestingly, trypanosomatid Vtc4 lacks an SPX domain, which is puzzling considering the essential role of the interaction of the SPX domain of Vtc2 or Vtc3 with the SPX domain of Vtc4 for the regulation of polyP polymerase function in yeasts (31). There is, however, indirect evidence that the synthesis of *Trypanosoma brucei* polyP is stimulated by PP-IPs. We determined that conditional knockout (KO) of *T. brucei* inositol polyphosphate multikinase, a precursor enzyme in the synthesis of PP-IPs (5-IP_7_), had less polyP and a reduced number, size, and electron density of acidocalcisomes (33).

In this work, we investigated whether the *T. cruzi* VTC complex is essential for parasite infectivity and whether it contains additional subunits required for its activity. We identified a novel component of the complex, which we termed TcVtc6, and found that the VTC complex is required for differentiation of amastigotes into trypomastigotes and for host cell egress.

## RESULTS

### Generation of *TcVtc1-KO* and *TcVtc4-SKO* cells and phenotypic changes

**Fig 1:**
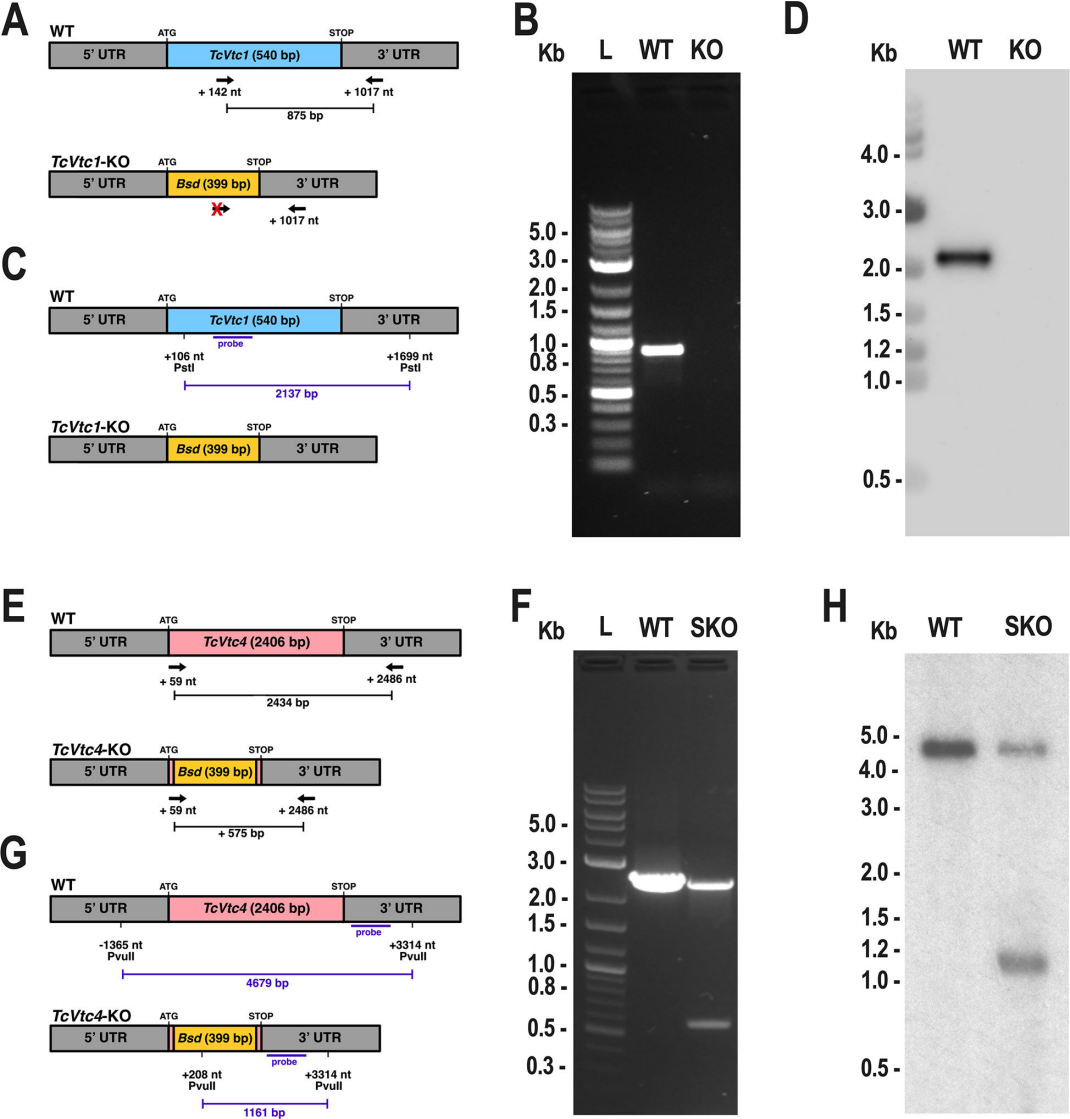
Generation of *TcVtc1*-KO and *TcVtc4*-SKO cell lines by CRISPR/Cas9. (**A**) Primers (arrows) used to verify gene replacement by PCR. The intact locus generates a PCR product of 875 bp, while the replaced locus does not generate a fragment. (**B**) PCR analysis showing that *TcVtc1* was ablated at its genomic locus. Lanes: L, 1 kb plus ladder; WT, wild type; and KO, *TcVtc1*-KO. (**C**) Strategy used for Southern blot analysis. Wild-type and *TcVtc1*-KO gDNA were digested with the PstI restriction enzyme. The blot was hybridized with a biotin-labeled probe corresponding to 375 bp of *TcVtc1* (nt +124 to +498). (**D**) Southern blot analysis of WT and *TcVtc1*-KO gDNA. (**E**) Primers (arrows) used to verify gene replacement by PCR. The intact locus generates a PCR product of 2,434 bp, while the disrupted locus generates a fragment of 575 bp. (**F**) PCR analysis showing that one *TcVtc4* allele was ablated at its genomic locus and the other was replaced in the genomic DNA of the SKO cell line. Lanes: L, 1 kb plus ladder; WT, wild type; and SKO, *TcVtc4*-SKO. (**G**) Strategy used for Southern blot analysis. Wild type and *TcVtc4*-SKO gDNA were digested with the PvuII restriction enzyme. The blot was hybridized with a ^32^P-labeled probe corresponding to 499 bp of *TcVTC4* 5′ UTR (nt +2471 to +2970). (**H**) Southern blot analysis of WT and *TcVtc4*-SKO gDNA.

To investigate the role of *TcVtc1* and *TcVtc4,* we attempted to generate *null* mutants of these genes following the CRISPR/Cas9 method in *T. cruzi* Y strain (34), which harbors a single copy of each gene for the haploid genome. As described in Materials and Methods, epimastigotes were transfected with specific molecular constructs for the constitutive expression of Cas9 nuclease and single-guide RNA (sgRNA) to target *TcVtc1* and *TcVtc4* genes. After selection with G418 and blasticidin, we obtained clonal populations from these cell lines by limiting dilution. Using specific sets of primers (Fig. 1A; see Table S1), we confirmed by PCR that the *TcVtc1* gene was ablated (Fig. 1B). Southern blot analysis confirmed that *TcVtc1* was absent in the genomic DNA of the KO cells (Fig. 1C and D). Attempts to generate *TcVtc4*-KO epimastigotes of *T. cruzi* using CRISPR/Cas9 genome editing were unsuccessful, suggesting that it is essential for normal growth. However, we were able to replace one allele with the DNA donor at the specific locus, as confirmed by PCR (Fig. 1E and F) and Southern blot (Fig. 1G and H) analyses.

**Fig 2:**
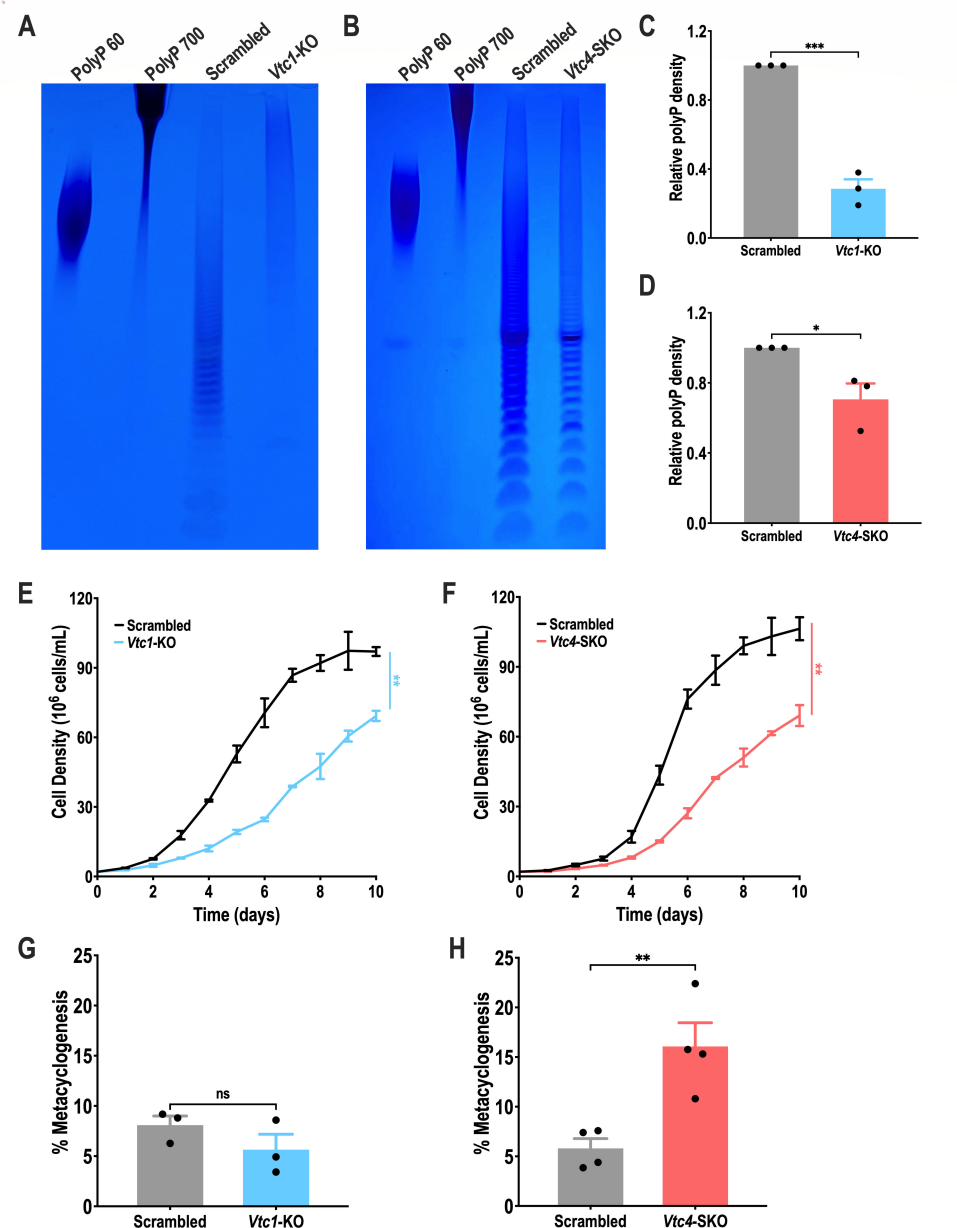
Phenotypic changes in *TcVtc1*-KO and *TcVtc4*-SKO. (**A**) Short-chain polyP was extracted from scrambled and *TcVtc1*-KO cells using the titanium dioxide bead enrichment method. PolyP_60_ and Poly_700_ were used as markers. (**B**) Short-chain polyP was extracted from scrambled and *TcVtc4*-SKO cells. PolyP_60_ and Poly_700_ were used as markers. (**C**) Densitometry of toluidine-stained polyP from scrambled and *TcVtc1*-KO cells. Values are means ± SEM (*n* = 3), ***P = 0.0002 by Student’s *t* test. (**D**) Densitometry of toluidine-stained polyP from scrambled and *TcVtc4*-SKO cells. Values are means ± SEM (*n* = 3), *P = 0.0316 by Student’s *t* test. (**E**) Growth of control (scrambled) and *TcVtc1*-KO epimastigotes in liver infusion tryptose (LIT) medium. Student’s *t* test was applied for the growth curve. Values are means ± SEM (*n* = 3), **P = 0.0014. (**F**) Growth of control (scrambled) and *TcVtc4*-SKO epimastigotes in LIT medium. Student’s *t* test was applied for the growth curve. Values are means ± SEM (*n* = 3), **P = 0.0038. (**G**) Percentage of metacyclic trypomastigotes in epimastigote cultures after incubation in triatomine artificial urine (TAU) 3AAG medium. Values are means ± SEM (*n* = 3). ns, no significant differences, by Student’s *t* test. (**H**) Percentage of metacyclic trypomastigotes in epimastigote cultures after incubation in TAU 3AAG medium. Values are means ± SEM (*n* = 4). **P < 0.007, by Student’s *t* test.

Both the *TcVtc1*-KO (Fig. 2A and C) and the *TcVtc4*-SKO (Fig. 2B and D) epimastigotes have less polyP and lower proliferation rates than the control cell line transfected with a scrambled sgRNA (Fig. 2E and F).

We also evaluated the ability of the mutants to undergo metacyclogenesis. *TcVtc1*-KO epimastigotes did not show significant differences compared to controls (Fig. 2G), whereas *TcVtc4*-SKO epimastigotes differentiated into metacyclic trypomastigotes at a higher proportion than control cells (Fig. 2H).

**Fig 3:**
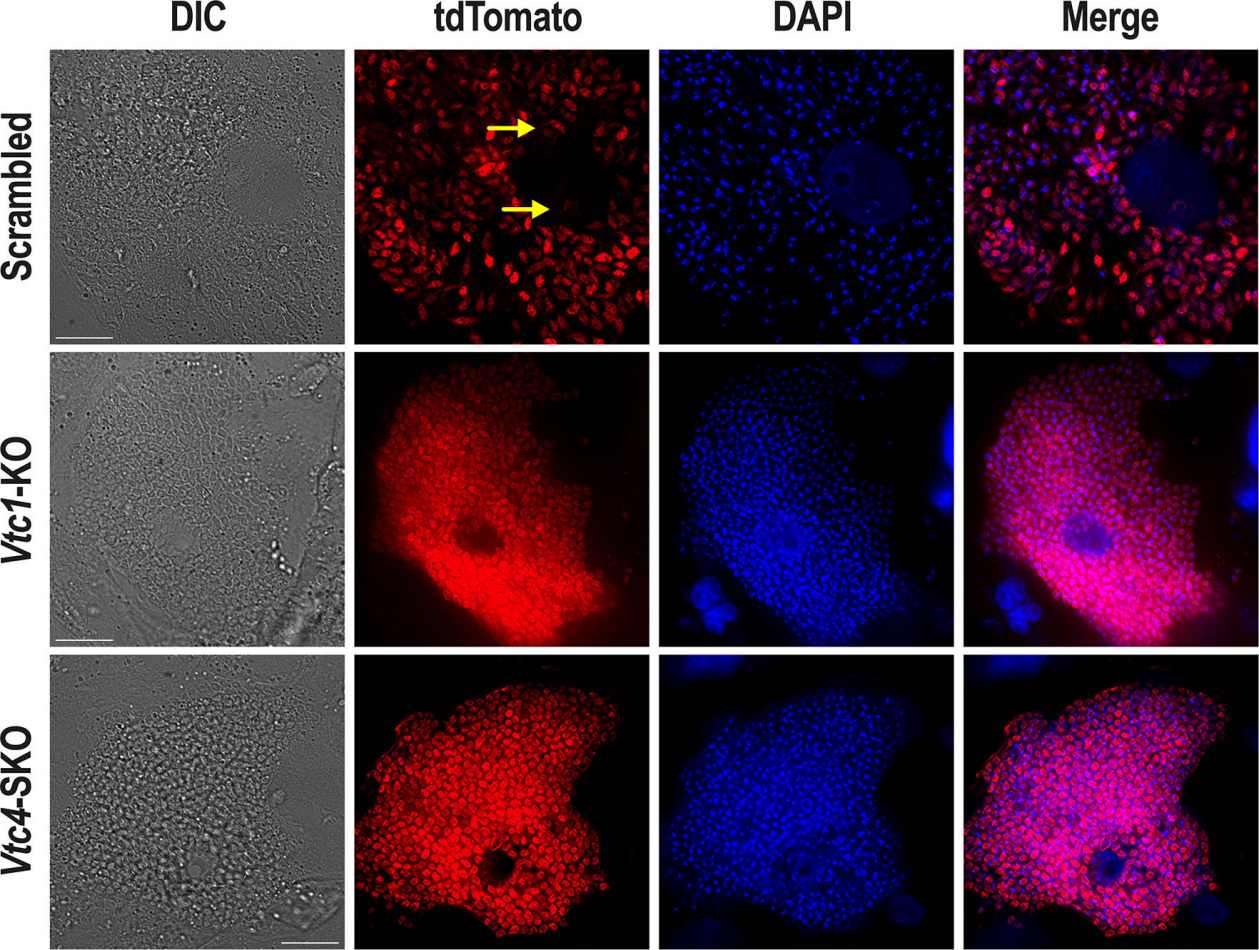
Infection of Vero cells with *TcVtc1*-KO and *TcVtc4*-SKO parasites. Vero cells were plated on coverslips and incubated with metacyclic trypomastigotes for 24 h, followed by washing with D-Hanks’ balanced salt solution. After 1 week (scrambled) and 3 weeks (*TcVtc1*-KO and *TcVtc4*-SKO), immunofluorescence assays were performed. In cells infected with scrambled parasites, amastigotes successfully differentiated into trypomastigotes (yellow arrows). In contrast, Vero cells infected with *TcVtc1*-KO or *TcVtc4*-SKO parasites remained filled with amastigotes, which failed to differentiate. DIC, differential interference contrast. TdTomato, cytosolic expression of tdTomato (red). DAPI staining of DNA (blue). Bars, 20 µm.

To assess the infectivity of *T. cruzi* in tissue culture cells, Vero cells were infected with metacyclic trypomastigotes to generate culture-derived trypomastigotes. In control cultures, culture-derived trypomastigotes were detected 4–5 days post-infection. In contrast, no trypomastigotes were observed following infection with either *TcVtc1*-KO or *TcVtc4*-SKO metacyclic trypomastigotes. In both cases, parasites successfully differentiated into amastigotes and escaped from the parasitophorous vacuole into the host cell cytosol. These parasites expressed tdTomato in their cytosol, which facilitated visualization by immunofluorescence assays (Fig. 3), while brightfield microscopy was used to monitor host cell infection and parasite replication (Fig. S1). Amastigotes continued to replicate for up to 3 weeks, ultimately leading to host cell rupture, but failed to undergo normal egress and differentiation into trypomastigotes. This phenotype was consistently observed throughout the infection time course.

### Conditional knockouts of *TcVtc1* and *TcVtc4*

We used a newly developed conditional knockout method based on the tetracycline-dependent activation of a hammerhead ribozyme (35) to obtain conditional KOs of both VTC subunits for comparative phenotypic analyses.

**Fig 4:**
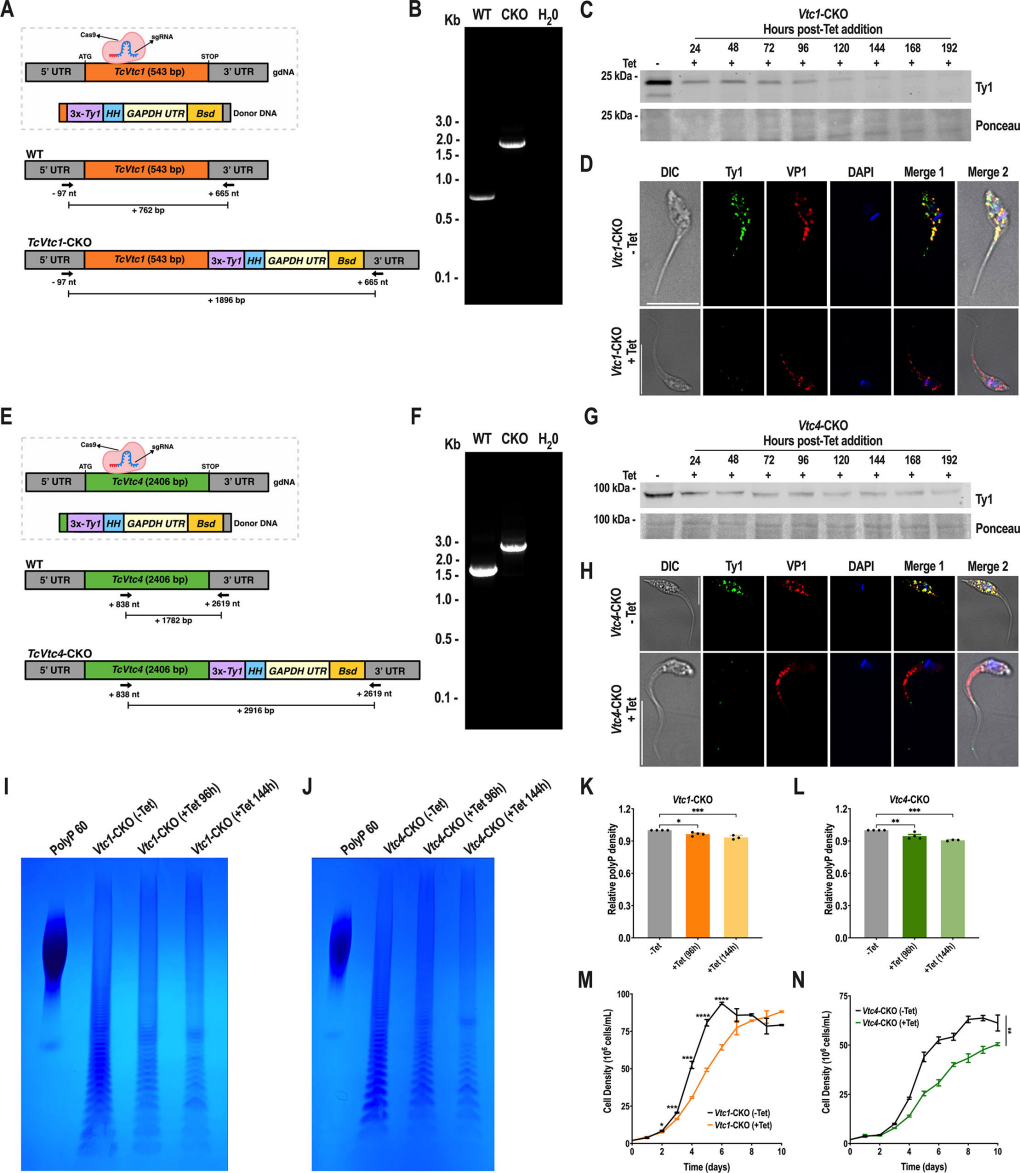
Conditional KO of *TcVtc1* and *TcVtc4*. (**A**) Primers (arrows) used to verify by PCR the endogenous C-terminal tagging of *TcVtc1* with an aptazyme cassette (composed of 3×Ty tag, ribozyme, *GAPDH* 3′ UTR, and *Bsd* gene) for conditional knockout (CKO) of *TcVtc1*. The intact locus generates a PCR product of 762 bp, while the modified locus generates a fragment of 1,896 bp. (**B**) PCR analysis for the validation of *TcVtc1* tagging showing the expected band for the control cell line (WT) and the *TcVtc1-*CKO cell line (CKO). Lanes: WT, wild type; CKO, *TcVtc1*-CKO; and H_2_O, PCR-negative control. (**C**) Western blot analysis of *TcVtc1-*CKO epimastigotes grown in the absence or presence of tetracycline. Anti-Ty1 antibody detects *TcVtc1-*CKO (expected size, 24 kDa). (**D**) Colocalization of Ty1-tagged TcVtc1 with the *T. cruzi* acidocalcisome marker vacuolar H^+^-pyrophosphatase (VP1) grown in the absence or presence of tetracycline. DIC, differential interference contrast. Ty1 (green), Ty1-tagged TcVtc1. VP1 (red), acidocalcisome marker. DAPI staining of DNA (blue). Bars, 20 µm. (**E**) Primers (arrows) used to verify by PCR the endogenous C-terminal tagging of *TcVtc4* with an aptazyme cassette (composed of 3×Ty tag, ribozyme, *GAPDH* 3′ UTR, and *Bsd* gene) for conditional knockout of *TcVtc4*. The intact locus generates a PCR product of 1,782 bp, while the modified locus generates a fragment of 2,916 bp. (**F**) PCR analysis for validation of *TcVtc4* tagging showing the expected band for the control cell line (WT) and the *TcVtc4-*CKO cell line (CKO). Lanes: WT, wild type; CKO, *TcVtc4*-CKO; and H_2_O, PCR-negative control. (**G**) Western blot analysis of *TcVtc4-*CKO epimastigotes grown in the absence or presence of tetracycline. Anti-Ty1 antibody detects *TcVtc4-Tet OFF* (expected size, 93 kDa). (**H**) Colocalization of Ty1-tagged TcVtc4 with the *T. cruzi* acidocalcisome marker vacuolar H^+^-pyrophosphatase (VP1) grown in the absence or presence of tetracycline. Ty1 (green), Ty1-tagged TcVtc4. VP1 (red), acidocalcisome marker. DAPI staining of DNA (blue). Bars, 20 µm. (**I**) Short-chain polyP was extracted from *TcVtc1-*CKO cells in the absence or presence of 5 μg/mL tetracycline for the indicated number of hours. PolyP_60_ was used as a marker. Background staining in the gel may affect the apparent band intensity. (**J**) Short-chain polyP was extracted from *TcVtc4-*CKO cells in the absence or presence of 5 μg/mL tetracycline for the indicated number of hours. PolyP_60_ was used as a marker. Background staining in the gel may affect the apparent band intensity. (**K**) Densitometry of toluidine-stained polyP from *TcVtc1-*CKO cells in the absence or presence of 5 μg/mL tetracycline for the indicated number of hours. Values are expressed as means ± SD (*n* = 4). *P ≤ 0.0109 and ***P ≤ 0.0004 by one-way ANOVA with Dunnett’s multiple comparison test. (**L**) Densitometry of toluidine-stained polyP from *TcVtc4-*CKO cells in the absence or presence of 5 μg/mL tetracycline for the indicated number of hours. Values are expressed as means ± SD (*n* = 4). **P ≤ 0.0054 and ***P ≤ 0.0003 by one-way ANOVA with Dunnett’s multiple-comparison test. (**M**) Growth of *TcVtc1-*CKO epimastigotes in the absence or presence of 5 μg/mL tetracycline for the indicated number of days. Student’s *t* test was applied for each day of the growth curve. (**N**) Growth of *TcVtc4-*CKO epimastigotes in the absence or presence of 5 μg/mL tetracycline for the indicated number of days. Student’s *t* test was applied for the growth curve. Values are means ± SEM (*n* = 3), **P = 0.0025.

We used the CRISPR/Cas9-mediated endogenous C-terminal tagging of epimastigotes (36) to introduce a 3×*Ty1* tag sequence into the *VTC* genes, followed by the tetracycline-inducible ribozyme (HH), a *GAPDH*-UTR, a blasticidin deaminase (*Bsd*) gene, and the *VTC* genes’ 3′ UTRs (Fig. 4A and E). After selection with G418 and blasticidin, we obtained clonal populations from these cell lines by limiting dilution. Using specific sets of primers (Fig. 4A and E; see Table S1), we confirmed by PCR that *TcVtc1* and *TcVtc4* genes were modified by the DNA donor cassette with the resistance marker at the specific locus (Fig. 4B and F). Addition of tetracycline resulted in downregulation of these genes and reduced protein levels, as shown by western blot (Fig. 4C and G) and immunofluorescence (Fig. 4D and H) analyses. This was accompanied by inhibition of polyP synthesis (Fig. 4I through L) and reduced cell proliferation (Fig. 4M and N). While statistically significant, the decrease in polyP accumulation in Fig. 4I through L was not as large compared to the effect of *Vtc1*-KO or *Vtc4*-SKO conditions (Fig. 2A through D).

### Search for divergent subunits of the TcVTC complex

**Fig 5:**
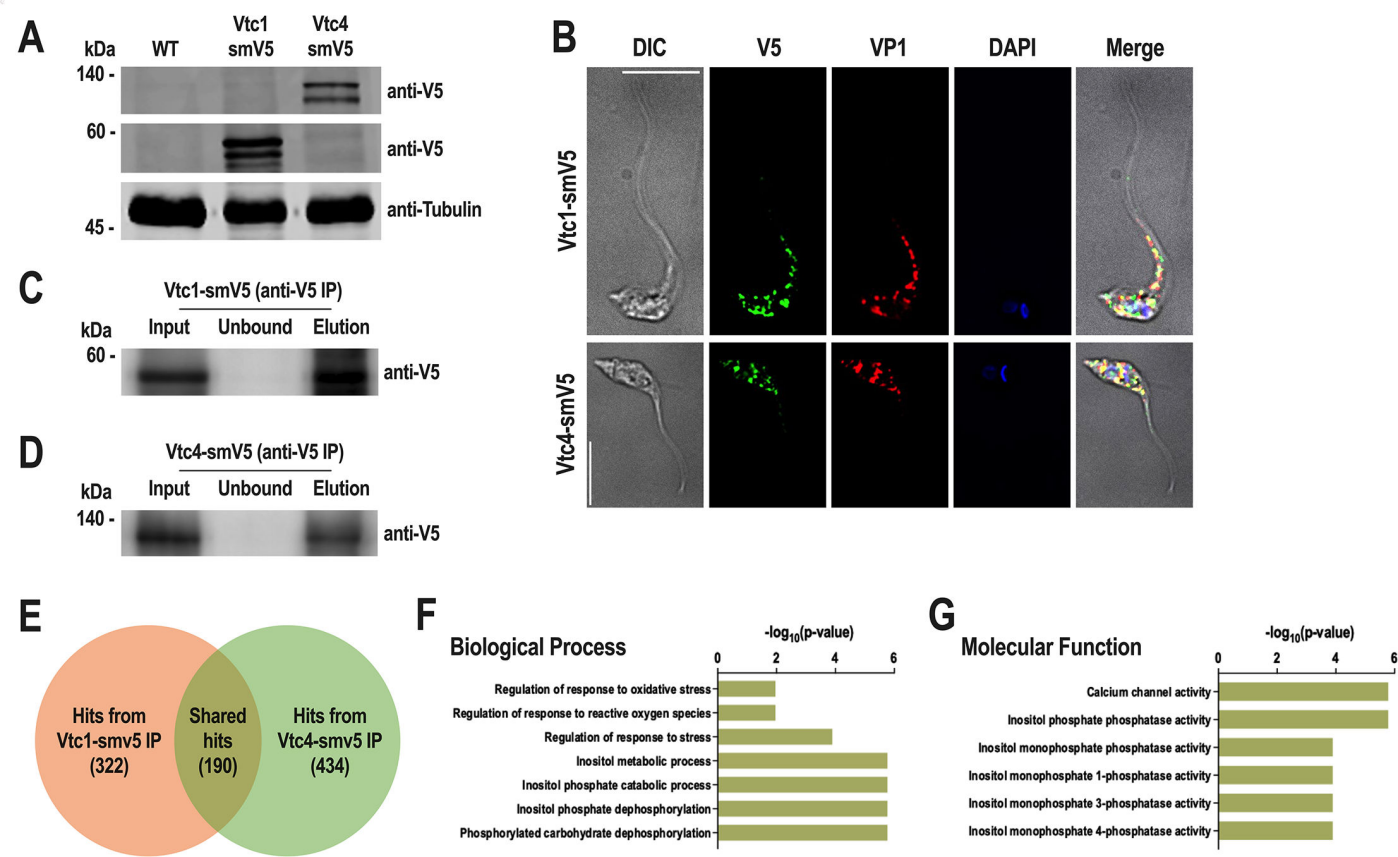
Endogenous C-terminal tagging of *TcVtc1* and *TcVtc4* to identify interacting partners by co-immunoprecipitation and mass spectrometry. (**A**) Western blot analysis of endogenously tagged TcVtc1-smV5 and TcVtc4-smV5 using anti-V5 antibodies. The predicted molecular masses of TcVtc1-smV5 and TcVtc4-smV5 are 66 and 135 kDa, respectively. Molecular weight markers are shown on the left. Tubulin was used as a loading control. (**B**) Immunofluorescence analysis showing colocalization of the green signal from smV5-tagged TcVtc1 or smV5-tagged TcVtc4 with VP1 (acidocalcisome marker). The merged images indicate colocalization (yellow). DIC, differential interference contrast. DAPI staining of DNA (blue). Bars, 5 µm. (**C**) Western blot analysis of co-immunoprecipitation using anti-V5 magnetic beads incubated with lysate from the smV5-tagged TcVtc1 cell line. Input (lysate), unbound, and eluted fractions were probed with anti-V5 antibodies, showing TcVtc1-smV5 captured in the elution fraction. (**D**) Western blot analysis of co-immunoprecipitation using anti-V5 magnetic beads incubated with lysate from the smV5-tagged TcVtc4 cell line. Input, unbound, and eluted fractions were probed with anti-V5 antibodies, showing TcVtc4-smV5 captured in the elution fraction. (**E**) Venn diagram of proteins enriched in TcVtc1-smV5 and TcVtc4-smV5 co-immunoprecipitations relative to control (fold change > 2). TcVtc1-smV5 captured 322 hits, TcVtc4-smV5 captured 434 hits, with 190 proteins shared between both. (**F**) Gene ontology (GO) enrichment analysis of biological processes for the 190 proteins shared between TcVtc1-smV5 and TcVtc4-smV5 co-immunoprecipitations (*P* < 0.06). (**G**) GO enrichment analysis of molecular functions for the 190 proteins shared between TcVtc1-smV5 and TcVtc4-smV5 co-immunoprecipitations (*P* < 0.06).

To identify potential interacting partners of the VTC complex in *T. cruzi*, we generated cell lines expressing TcVtc1 or TcVtc4 tagged at their C-terminus with smV5. The smV5 tag sequence was inserted at the 3′ end of each gene, with donor constructs containing the tag, an intergenic region (*IgR*), a puromycin resistance marker (*PAC*, encoding puromycin N-acetyltransferase, 600 bp), and two 100-bp homology arms. Western blot analysis verified the expression of the tagged proteins (Fig. 5A), and immunofluorescence demonstrated colocalization of TcVtc1-smV5 and TcVtc4-smV5 with the acidocalcisome marker TcVP1 (Fig. 5B). Co-immunoprecipitation using anti-V5 magnetic beads captured TcVtc1-smV5 and TcVtc4-smV5 from lysates, with wild-type (WT) lysates processed in parallel as controls (Fig. 5C and D). Mass spectrometry analysis identified 322 proteins enriched in TcVtc1-smV5 samples and 434 proteins enriched in TcVtc4-smV5 samples relative to control (fold change > 2), with 190 proteins shared between both data sets (Fig. 5E). Volcano plots were generated to visualize proteins with fold change >2 relative to WT (Fig. S2A for TcVtc1 and Fig. S2B for TcVtc4), highlighting the most enriched candidates. As only a single replicate per condition was analyzed, these results were considered exploratory, and fold change values were used to guide candidate selection rather than statistical conclusions.

Gene ontology enrichment analysis of these shared proteins revealed overrepresented molecular functions, including inositol phosphate phosphatase activity and calcium channel activity (Fig. 5F and G). Among the most highly enriched candidates in both data sets was TryTripDB ID: TcYC6_0091170, a 34 kDa hypothetical protein conserved only in kinetoplastids.

*In silico* analysis predicted a single transmembrane domain, and previous studies have described that its *T. brucei* ortholog (TryTripDB ID: Tb927.4.860) is an acidocalcisome-localized protein (37, 38). Further structural analysis using COFACTOR2 (39) identified 10 structural analogs, 5 of which were related to VTC proteins, making it an interesting candidate for further study. Based on these observations, TcYC6_0091170 was selected for further characterization and is hereafter referred to as TcVtc6.

### Novel subunit of the *T. cruzi* VTC complex

**Fig 6:**
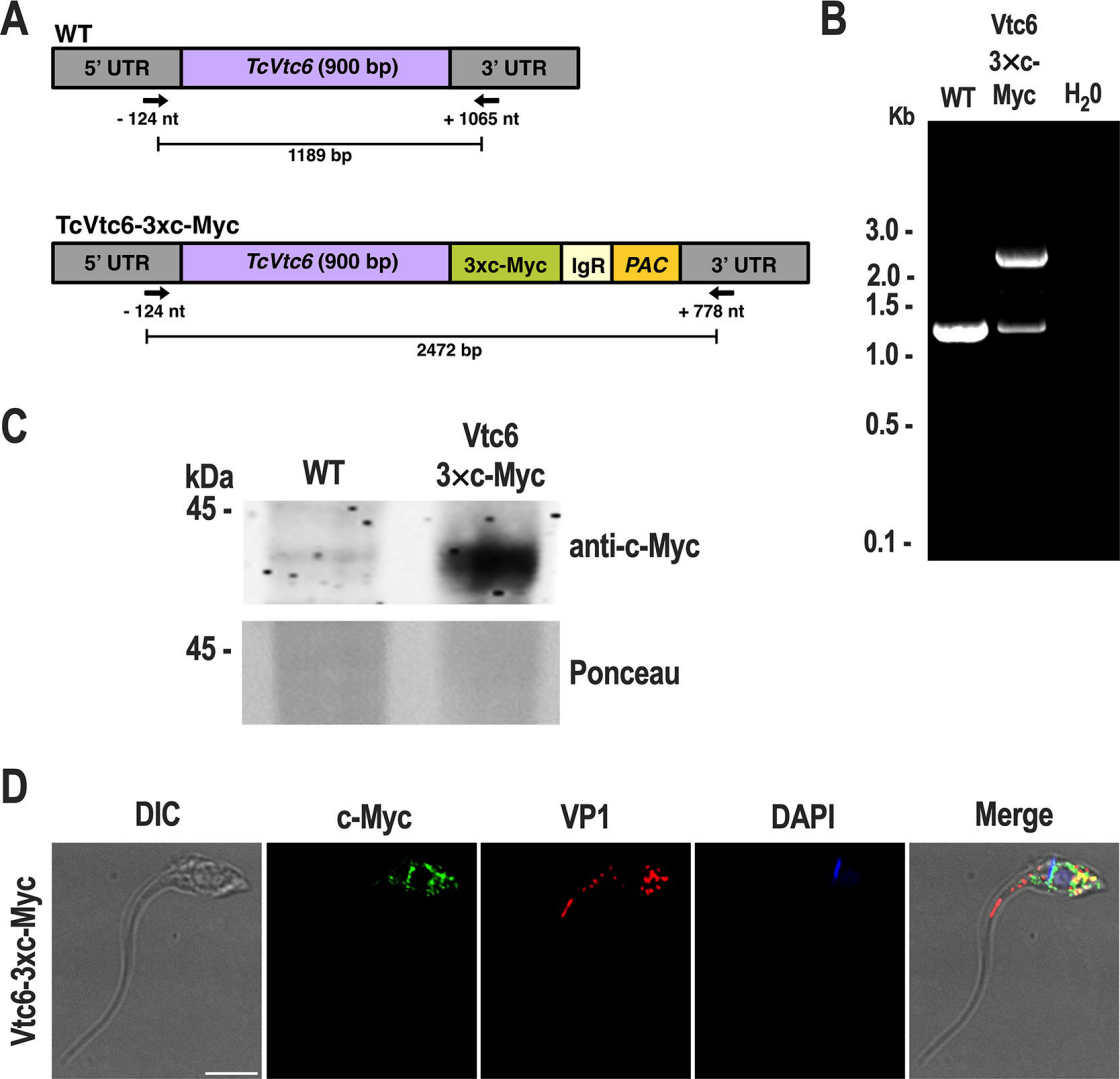
Endogenous C-terminal tagging of *TcVtc6* by CRISPR/Cas9. (**A**) Primers (arrows) used to verify by PCR the endogenous C-terminal tagging of *TcVtc6*. The intact locus generates a PCR product of 1,189 bp, while the modified locus generates a fragment of 2,472 bp. (**B**) PCR analysis for validation of *TcVtc6* tagging showing expected bands for control cell line (WT) and *TcVtc6*-3×c-Myc cell line. Lanes: WT, wild type; *TcVtc6*-3×c-Myc, tagged *TcVtc6*; and H_2_O, PCR-negative control. (**C**) Western blot analysis of WT and *TcVTC6*-3×c-Myc epimastigotes using the monoclonal antibody against c-Myc tag. The predicted protein molecular mass for *TcVtc6*-3×c-Myc is 39 kDa. Molecular markers are on the left. Ponceau staining was used as a loading control. (**D**) Immunofluorescence analysis showing colocalization of the green signal from 3×c-Myc-tagged TcVtc6 with VP1 (acidocalcisome marker). The merged images indicate colocalization (yellow). DIC, differential interference contrast. DAPI staining of DNA (blue). Bar, 5 µm.

To determine its subcellular localization, *TcVtc6* was endogenously tagged using CRISPR/Cas9, as described previously (36). A nucleotide sequence encoding a 3×c-Myc tag was inserted at the 3′ end of *TcVtc6*. The donor sequences contained the tag sequence, an intergenic sequence (*IgR*), the marker for resistance to puromycin (*PAC*, 600 bp), and two 100-bp homology arms (Fig. 6A). Successful tagging was confirmed by PCR (Fig. 6B) and western blot analysis (Fig. 6C). Immunofluorescence analysis showed that TcVtc6 colocalizes with the acidocalcisome marker TcVP1 (40) (Fig. 6D).

**Fig 7:**
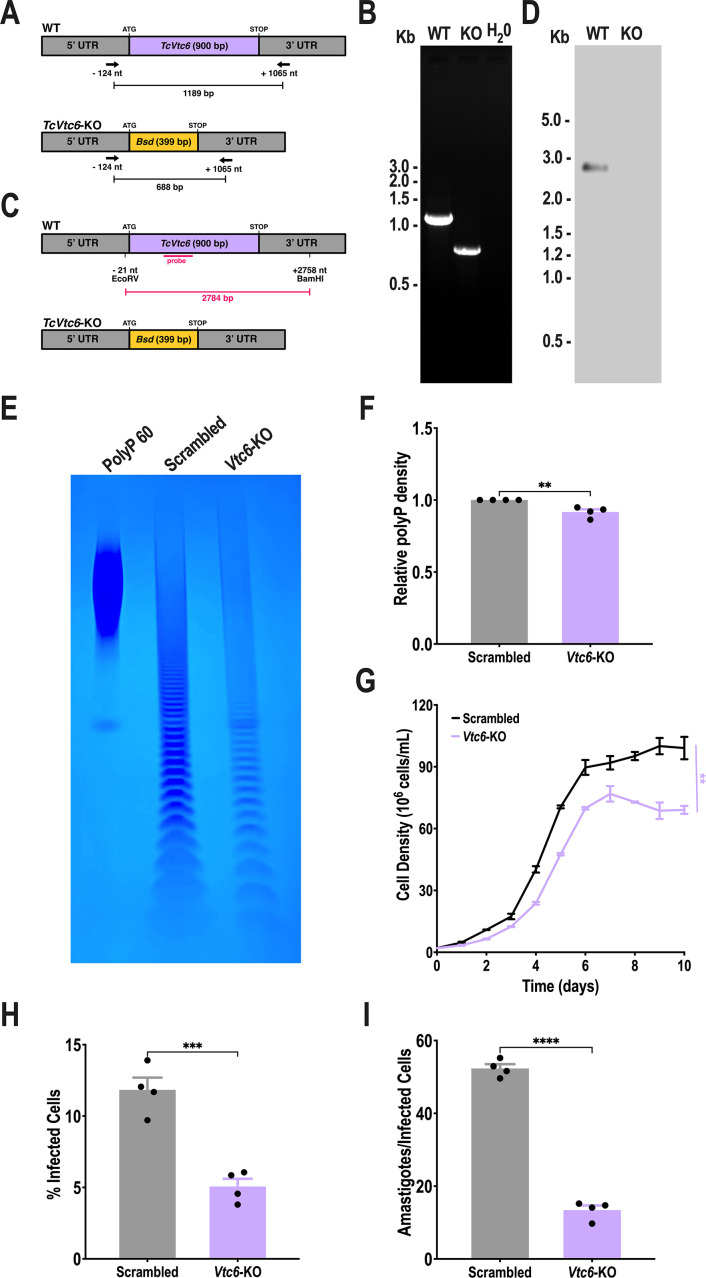
Generation and phenotypic analysis of *TcVtc6*-KO. (**A**) Primers (arrows) used to verify gene replacement by PCR. The intact locus generates a product of 1,189 bp, while the replaced locus generates a fragment of 688 bp. (**B**) PCR analysis showing that *TcVtc6* was ablated at its genomic locus and replaced in the genomic DNA of the KO cell line. Lanes: WT, wild type; KO, *TcVtc6*-KO; and H_2_O, PCR-negative control. (**C**) Strategy used for Southern blot analysis. Wild-type and *TcVtc6*-KO gDNA were digested with EcoRV and BamHI restriction enzymes. The blot was hybridized with a biotin-labeled probe corresponding to 411 bp (nt +27 to +437) of *TcVtc6*. (**D**) Southern blot of wild type and *TcVtc6*-KO gDNA showing probe hybridization. (**E**) Short-chain polyP was extracted from scrambled and *TcVtc6*-KO cells. PolyP_60_ was used as a marker. (**F**) Densitometry of toluidine-stained polyP from scrambled and *TcVtc6*-KO cells. Values are means ± SEM (*n* = 4). **P = 0.0044 by Student’s *t* test. (**G**) Growth of control (scrambled) and *TcVtc6*-KO epimastigotes in liver infusion tryptose medium. Student’s *t* test was applied to the growth curve. Values are means ± SEM (*n* = 3), **P = 0.0011. (**H**) *TcVtc6*-KO trypomastigote infection of Vero cells at 4 h post-infection was significantly inhibited. Values are expressed as means ± SD (*n* = 4), ***P = 0.0005 by Student’s *t* test. (**I**) The number of intracellular amastigotes per infected host cell observed 48 h post-infection was also significantly reduced. Values are expressed as means ± SD (*n* = 4), ****P < 0.0001 by Student’s *t* test.

To investigate the role of *TcVtc6,* we generated *null* mutants of this gene following the CRISPR/Cas9 method in *T. cruzi* Y strain as described above. We used epimastigotes transfected with specific molecular constructs for the constitutive expression of Cas9 nuclease and sgRNA to target the *TcVtc6* gene. After selection with G418 and blasticidin, we obtained clonal populations from these cell lines by limiting dilution. Using specific sets of primers (Fig. 7A; see Table S1), we confirmed by PCR that the *TcVtc6* gene was ablated and replaced by the DNA donor cassette with the resistance marker at the specific locus (Fig. 7B). Southern blot analysis confirmed that *TcVtc6* was absent in the genomic DNA of the KO cells (Fig. 7C and D). *TcVtc6*-KO epimastigotes have less polyP (Fig. 7E and F) and lower proliferation rates than the control cell line transfected with a scrambled sgRNA (Fig. 7G). We infected Vero cells with tissue culture-derived trypomastigote stages and measured both the ability of trypomastigotes to infect host cells and the replication of intracellular amastigotes, as described in Materials and Methods. While the effect of *TcVtc6*-KO was mild regarding polyP accumulation (Fig. 7E and F), it was rather profound regarding the ability of trypomastigotes to infect host cells (Fig. 7H) or the replication of intracellular amastigotes (Fig. 7I).

**Fig 8:**
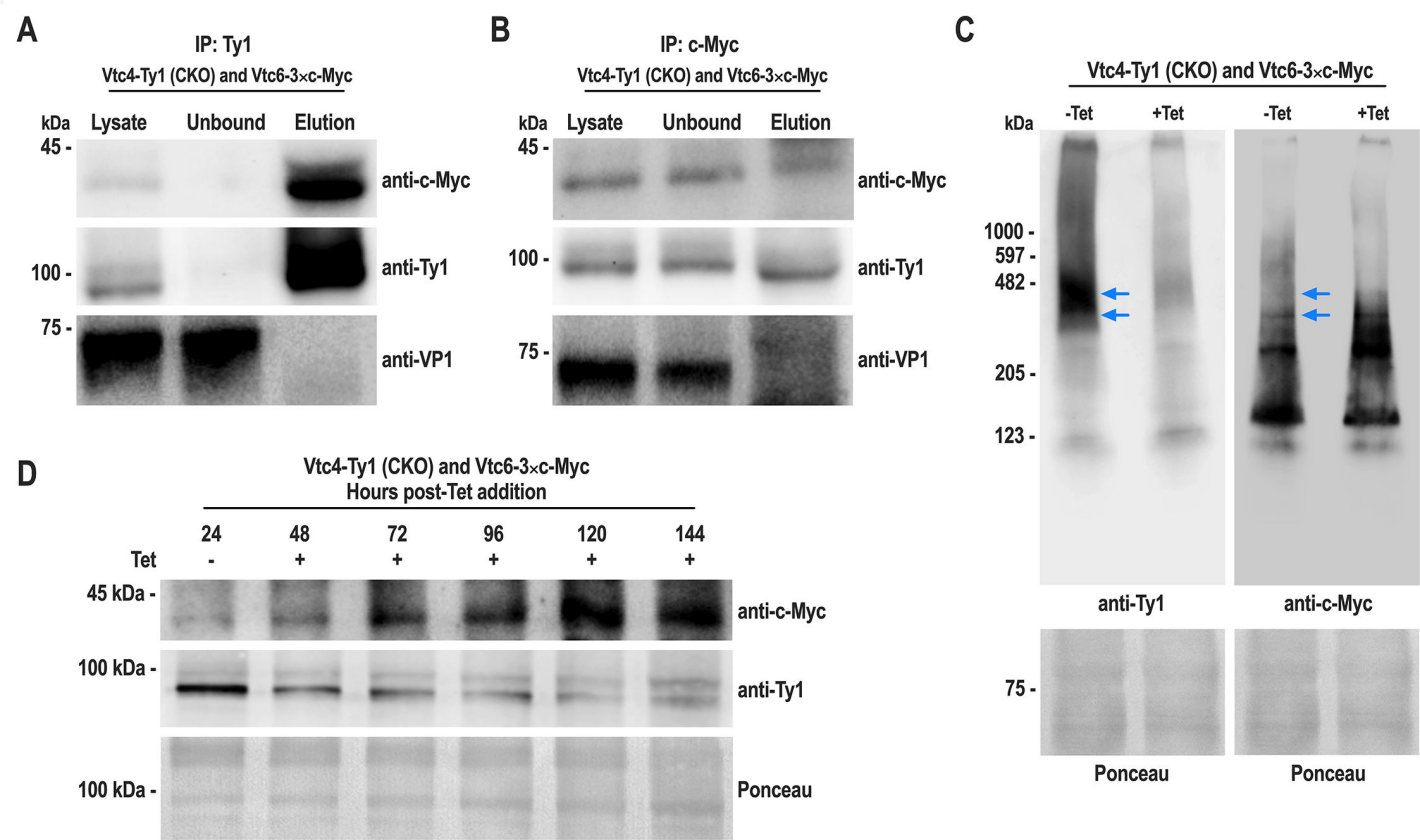
Analysis of TcVtc6 interaction with TcVtc4 in the TcVtc4-Ty1 conditional knockdown cell line. (**A**) Reciprocal co-immunoprecipitation of TcVtc4-Ty1 and TcVtc6-3×c-Myc. Lysate from the TcVtc4-Ty1/TcVtc6-3×c-Myc cell line was incubated with anti-c-Myc magnetic beads. Input (5% of total lysate), unbound, and eluted fractions were analyzed by Western blot using anti-c-Myc and anti-Ty1 antibodies. Anti-VP1 was used as a non-interacting protein control to monitor non-specific binding. (**B**) Reciprocal co-immunoprecipitation of TcVtc6-3×c-Myc and TcVtc4-Ty1. Lysate from the TcVtc4-Ty1/TcVtc6-3×c-Myc cell line was incubated with anti-Ty1 magnetic beads. Input, unbound, and eluted fractions were analyzed by Western blot using anti-Ty1 and anti-c-Myc antibodies. Anti-VP1 was used as a non-interacting protein control to monitor non-specific binding. (**C**) Blue Native PAGE analysis of TcVtc6-3×c-Myc and TcVtc4-Ty1 protein complexes in the TcVtc4-Ty1/TcVtc6-3×c-Myc cell line in the presence or absence of tetracycline. Lysates were separated under native conditions and analyzed by Western blot using anti-c-Myc and anti-Ty1 antibodies. Ponceau staining was used as a loading control. The same membrane was sequentially probed with anti-Ty1 (left) and anti-c-Myc (right) antibodies; therefore, the identical Ponceau staining image is presented beneath both panels. Blue arrows indicate the bands corresponding to the potential complexes. (**D**) Western blot analysis of TcVtc4-Ty1 and TcVtc6-3×c-Myc expression over time in the TcVtc4-Ty1/TcVtc6-3×c-Myc cell line treated with 5 μg/mL tetracycline. Samples were collected at 24, 48, 72, 96, 120, and 144 h post-treatment and probed with anti-Ty1 and anti-c-Myc antibodies. Anti-VP1 was used as a loading control.

To confirm the interaction of TcVtc6 with other VTC complex components identified in the TcVtc1 and TcVtc4 co-immunoprecipitation and mass spectrometry analysis, we tagged TcVtc6 with a 3×c-Myc epitope in the *TcVtc4*-Ty1 conditional knockdown cell line (Fig. 4E through N). Reciprocal co-immunoprecipitation experiments using anti-c-Myc and anti-Ty1 magnetic beads demonstrated that TcVtc6 and TcVtc4 associate in the same protein complex (Fig. 8A and B). In both pull-downs, the interacting partner was detected in the elution fraction at the expected molecular weight. In contrast, the control protein VP1 was detected in the input and unbound fractions but not in the elution fraction, indicating that non-specific proteins were not retained by the beads. Blue Native PAGE analysis of these cell lysates further confirmed the presence of TcVtc6 and TcVtc4 in high-molecular-weight protein complexes and revealed that depletion of TcVtc4 upon tetracycline treatment led to a reduction of these higher-order complexes (Fig. 8C). Time-course analysis of *TcVtc4* knockdown cells showed that TcVtc6 protein levels increased over 144 h in the presence of tetracycline, whereas TcVtc4 levels decreased as expected (Fig. 8D). Similarly, tagging TcVtc1 with smV5 in the TcVtc4-Ty1 conditional knockdown cell line revealed that TcVtc1 protein levels also increased upon TcVtc4 depletion, accompanied by loss of higher-molecular-weight complexes under native conditions (Fig. S3A and B). These results indicate that both TcVtc6 and TcVtc1 are part of TcVtc4-containing complexes and are upregulated when TcVtc4 expression is reduced.

## DISCUSSION

Our work has revealed the essentiality of *T. cruzi* VTC complex for the synthesis of polyP, for the normal proliferation of its epimastigote stage, for differentiation of amastigotes into trypomastigotes within host cells, and for parasite egress from the host cells. In addition, coimmunoprecipitation studies followed by proteomic analyses led to the discovery of TcVtc6, a novel subunit of the *T. cruzi* VTC complex. TcVtc6 is involved in polyP synthesis, as its deletion reduces polyP levels. TcVtc6 interacts with TcVtc4, forming a large protein complex detectable by Blue Native PAGE and immunoblotting of the subunits.

Metacyclic trypomastigotes obtained by *in vitro* differentiation of *TcVtc1*-KO and *TcVtc4*-SKO epimastigotes were able to invade host cells, differentiate to intracellular amastigotes, escape from the parasitophorous vacuole, and replicate within host cells but were unable to convert back to trypomastigotes and egress. The lack of transformation of amastigotes to cell-derived trypomastigotes prevented more quantitative comparative studies of wild-type and mutant parasites. The reason why amastigotes could not differentiate back to trypomastigotes and egress from the host cells is unknown, but a similar inhibition of amastigote to trypomastigote conversion and parasite egress has been reported upon disruption of active *trans*-sialidase genes (41).

Early DNA microarray studies identified the *PHM1-PHM4* genes in *S. cerevisiae,* encoding proteins proposed to be involved in polyP synthesis, as demonstrated by the lack of detectable polyP in *phm3*Δ and *phm4*Δ mutants or in *phm1*Δ/*phm*2Δ double mutants (42). The *PHM1-PHM4* genes were independently identified and named *VTC1-4* (*VTC1/PHM4, VTC2/PHM1, VTC3/PHM2,* and *VTC4/PHM3*) (43), and in 2009, it was demonstrated that Vtc4 is the catalytic subunit of this polyP polymerase complex (27). More recent studies have described Vtc5 as an additional component of the complex in *S. cerevisiae* that could regulate its activity independently of PP-IPs (29, 44, 45). However, only yeasts appear to possess all these Vtc1–5 subunits. Except for Vtc1, all Vtc subunits from yeasts (Vtc2, Vtc3, Vtc4, and Vtc5) possess an SPX domain, which is important for the stimulation of their catalytic activity by PP-IPs. However, trypanosomatids lack Vtc2, Vtc3, and Vtc5, while their Vtc4 and Vtc6 subunits lack an SPX domain. The absence of these SPX domains in the VTC components of trypanosomatids implies that there cannot be an SPX-dependent regulation of the VTC complex by PP-IPs.

PP-IPs have been shown to stimulate only one SPX domain-controlled process in trypanosomatids: acidocalcisome P_i_ efflux by the Na^+^/P_i_ symporter Pho91 from *T. brucei* (TbPho91), which is stimulated by 5-diphosphoinositol pentakisphosphate (5-IP7) (46). The lack of SPX domain-containing VTC subunits in trypanosomatids would prevent the paradoxical simultaneous SPX-dependent stimulation of polyP synthesis and P_i_ release from the same vacuoles.

Studies in other protists have identified other putative components of the VTC complex. *Chlamydomonas reinhardtii*, like trypanosomatids, possesses Vtc1 and Vtc4 orthologs but lacks Vtc2, Vtc3, and Vtc5 homologs. It also has other sequences that encode proteins with similarities to Vtc1 (*Cre01.g402812, Cre10.g461500,* and *Cre13.g583200*) and a gene encoding a putative accessory component of the VTC complex (*Cre01.g005500*) (47). These genes, like *Vtc1* and *Vtc4,* have reduced transcript levels after 24 h of addition of PO_4_^3−^ to a P_i_-deficient medium (48). The apicomplexan parasite *Toxoplasma gondii* possesses orthologs to Vtc2 (23) and Vtc4 (49) but not to Vtc1, Vtc3, and Vtc5. When this paper was in preparation, three putative binding partners (LtVBP1–LtVBP3) of the VTC complex were described in *Leishmania tarentolae* (50). These binding partners colocalize and interact with the VTC complex in acidocalcisomes, as suggested by confocal microscopy, pulldown assays, and AlphaFold 3 structural predictions (50). LtVBP3 is a homolog of TcVtc6, further supporting our work. Interestingly, LtVBP3 was predicted to bind indirectly to the VTC complex (50), which could explain the relatively weak pull-down shown in Fig. 8B when the immunoprecipitation was done with anti-c-Myc, suggesting a transient or indirect interaction. Another possibility is that the interaction between TcVtc6 and the beads was not strong enough, and part of the proteins were lost during the washing due to a technical issue with the beads under the conditions in which the experiments were carried out. Taken together, these results suggest that other components different from those present in yeasts might be present in the VTC complex of other eukaryotes.

In conclusion, we have identified a novel subunit of the trypanosomatid VTC complex and characterized the phenotypic consequences of knockout and downregulation of its subunits, highlighting their essential roles in polyphosphate synthesis, differentiation, and parasite infectivity.

## MATERIALS AND METHODS

### Culture methods

*T. cruzi* (Y strain) epimastigotes were maintained at 28°C in liver infusion tryptose (LIT) medium (51) supplemented with 10% newborn calf serum (NCS), penicillin (100 U/mL), and streptomycin (100 µg/mL). Mutant cell lines were maintained in a medium containing 250 µg/mL G418, 10 µg/mL blasticidin, or 5 µg/mL puromycin. Epimastigote growth rates were determined by counting cells every 24 h using a Coulter Counter (Beckman Coulter). Tissue culture-derived trypomastigotes were obtained from Vero cells infected with metacyclic trypomastigotes as described below. Trypomastigotes were collected from the culture medium of infected host cells using a modification of the method of Schmatz and Murray (52) as previously described (51). Vero cells were cultured in RPMI medium supplemented with 10% fetal bovine serum and maintained at 37°C with 5% CO_2_.

### Cell transfection

Transfections were performed as previously described (53). *Trypanosoma cruzi* Y strain epimastigotes (4 × 10⁷ cells) were washed with phosphate-buffered saline (PBS; pH 7.4) at room temperature and transfected in ice-cold CytoMix (120 mM KCl, 0.15 mM CaCl_₂_, 10 mM K_₂_HPO_₄_, 25 mM HEPES, 2 mM EDTA, and 5 mM MgCl_₂_; pH 7.6) containing 25 µg of each plasmid or DNA donor construct. Electroporations were performed in 4 mm gap cuvettes using three pulses (1,500 V, 25 µF) delivered by a Gene Pulser Xcell Electroporation System (Bio-Rad). Stable cell lines were established and maintained under drug selection with the appropriate antibiotics (250 µg/mL G418, 10 µg/mL blasticidin, and/or 5 µg/mL puromycin). Transfectant epimastigotes were cultured in LIT medium supplemented with 20% heat-inactivated NCS. Parasite clones were obtained by limiting dilution.

### Endogenous C-terminal tagging of *TcVtc1, TcVtc4,* and *TcVtc6*

Endogenous C-terminal tagging of *TcVtc1, TcVtc4*, and *TcVtc6* was performed using a CRISPR/Cas9-mediated strategy. *T. cruzi* Y strain epimastigotes constitutively expressing T7 RNA polymerase and Cas9 were co-transfected with sgRNA templates generated by PCR (Table S1, primers 3 and 4 for Vtc1; primers 4 and 16 for Vtc4; and primers 4 and 33 for Vtc6) and donor DNA cassettes amplified from pMOTag23M (54) or pMOTag2mV (55) vectors (Table S1, primers 1 and 2 for Vtc1; primers 14 and 15 for Vtc4; and primers 31 and 32 for Vtc6). Protospacer sequences were selected using EuPaGDT (Eukaryotic Pathogen CRISPR guide RNA Design Tool), and sgRNAs were designed to target regions near the 3′ end of the *TcVtc1, TcVtc4,* or *TcVtc6* ORFs. Transfectants were selected with puromycin. Correct endogenous tagging was confirmed by PCR using genomic DNA and gene-specific primers (Table S1, primers 10 and 11 for Vtc1; primers 19 and 20 for Vtc4; and primers 34 and 35 for Vtc6) and by western blot analysis.

### Knockout of *TcVtc1*

Knockout of *TcVtc1* was generated using the *T. cruzi* T7RNAP/Cas9 system as previously described (56). Chimeric single-guide RNA template targeting *TcVtc1* (TryTripDB ID: TcYC6_0018480) was amplified by PCR (Table S1, primers 4 and 7). The protospacer sequence was selected using EuPaGDT (Eukaryotic Pathogen CRISPR guide RNA Design Tool), and the protospacer, together with the T7 polymerase promoter sequence, was incorporated into the forward primer, using a common reverse primer for sgRNA amplification. A donor DNA cassette designed to promote homologous-directed repair and replacement of the *TcVtc1* ORF was generated by PCR using the primers listed in Table S1 (primers 8 and 9), with pBSD as the template. The forward primer contained a 40-nt homologous region corresponding to the 5′ UTR of TcVtc6, followed by 20 nucleotides of the plasmid backbone, including the start codon (5′-GCCGCGGGAATTCGATTATG-3′). The reverse primer contained a 37-nt homologous region corresponding to the 3′ UTR of *TcVtc1* followed by 23 nucleotides of the plasmid backbone and the final four nucleotides of the antibiotic resistance gene, including the stop codon (5′-CGCGAATTCACTAGTGATTTCAC-3′). *T. cruzi* T7RNAP/Cas9 epimastigotes were co-transfected with the sgRNA template (25 µg) and donor DNA cassette (25 µg). Transfected parasites were selected for 4 weeks in the presence of G418 and blasticidin. Disruption of the *TcVtc1* gene was confirmed by PCR using genomic DNA from mutant parasites (Table S1, primers 10 and 11).

### Knockout of *TcVtc4*

Chimeric sgRNA sequence to target the *TcVtc4* gene (TripDB ID: TcYC6_0036060) was PCR amplified (Table S1, primers 21 and 22) from plasmid pUC_sgRNA, as previously described (34). The selection of the protospacer was performed using EuPaGDT. The protospacer sequence was included in the forward primer, while using a common reverse primer for sgRNA amplification. The sgRNA orientation was verified by PCR using the specific TcVtc4-sgRNA forward primer and the HX1 reverse primer (Table S1, primers 21 and 25). These primers also contained a BamHI restriction site for cloning into Cas9/pTREX-n to generate the *TcVtc4*sgRNA/Cas9/pTREX-n construct. Positive clones that generate a 190-bp PCR fragment were also sequenced. A scrambled sgRNA (Scrambled-sgRNA/Cas9/pTREX-n) was used as a control. A DNA donor cassette designed to promote homologous-directed repair and replacement of *TcVtc4* ORF was obtained by PCR using a set of long primers (ultramers) containing 120 nucleotides, from which 100 nucleotides correspond to the first 100 nt (forward ultramer) and the last 100 nt (reverse ultramer) of *TcVtc4* ORF, and 20 nt annealing on the blasticidin gene (Table S1, primers 23 and 24). *TcVtc4*-sgRNA/Cas9/pTREX-n construct (25 µg) and linear blasticidin cassette (25 µg) were used to transfect epimastigotes. After 5 weeks of selection with 250 µg/mL G418 and 10 µg/mL blasticidin, *TcVtc4* gene replacement was verified by PCR using primers 19 and 20 (Table S1).

### Knockout of *TcVtc6*

Knockout of *TcVtc6* was generated using the *T. cruzi* T7RNAP/Cas9 system as previously described (56). Chimeric single-guide RNA template targeting *TcVtc6* (TryTripDB ID: TcYC6_0091170) was amplified by PCR (Table S1, primers 4 and 28). The protospacer sequence was selected using EuPaGDT, and the protospacer, together with the T7 polymerase promoter sequence, was incorporated into the forward primer, using a common reverse primer for sgRNA amplification. A donor DNA cassette designed to promote homologous-directed repair and replacement of the *TcVtc6* ORF was generated by PCR using primers listed in Table S1 (primers 29 and 30), with pBSD as template. The forward primer contained a 40-nt homologous region corresponding to the 5′ UTR of TcVtc6, followed by 20 nucleotides of the plasmid backbone, including the start codon (5′-GCCGCGGGAATTCGATTATG-3′). The reverse primer contained a 37-nt homologous region corresponding to the 3′ UTR of *TcVtc6,* followed by 23 nucleotides of the plasmid backbone and the final four nucleotides of the antibiotic resistance gene, including the stop codon (5′-CGCGAATTCACTAGTGATTTCAC-3′). *T. cruzi* T7RNAP/Cas9 epimastigotes were co-transfected with the sgRNA template (25 µg) and donor DNA cassette (25 µg). Transfected parasites were selected for 4 weeks in the presence of G418 and blasticidin. Disruption of the *TcVtc6* gene was confirmed by PCR using genomic DNA from mutant parasites (Table S1, primers 34 and 35).

### Conditional knockout of *TcVtc1* and *TcVtc4*

Conditional downregulation of *TcVtc1* and *TcVtc4* was achieved using the small hammerhead aptazyme-regulated knockdown system for *T. cruzi*, which employs modified hammerhead ribozymes (35). *TcVtc1* and *TcVtc4* aptazyme tagging cassettes, comprising a 3×Ty-tag, *Tet-OFF* aptazyme sequence, *GAPDH 3′ UTR*, and the blasticidin resistance gene (*Bsd),* were generated by PCR using the pMiniTrex-mCherry-aptazyme plasmid (35) as template and gene-specific primers (Table S1, primers 5 and 6 for Vtc1, and primers 17 and 18 for Vtc4). Forward and reverse primers contained 50-bp homology arms corresponding to regions near the 3′ end of each ORF, followed in frame by 23 nucleotides of the pMiniTrex-mCherry-aptazyme backbone. In addition, chimeric sgRNA templates targeting *TcVtc1* (TryTripDB ID: TcYC6_0018480) and *TcVtc4* (TryTripDB ID: TcYC6_0036060) were generated by PCR (Table S1, primers 3 and 4 for Vtc1, and primers 4 and 16 for Vtc4). Protospacer sequences were selected using EuPaGDT, and the protospacer, along with the T7 promoter sequence, was incorporated into the forward primer, using a common reverse primer for sgRNA amplification. Gel-purified aptazyme-tagging cassettes (25 µg) and the corresponding sgRNA templates (25 µg) were co-transfected into *T. cruzi* T7RNAP/Cas9 epimastigotes to generate *TcVtc1 Tet-OFF* (*TcVtc1*-CKO) and *TcVtc4 Tet-OFF* (*TcVtc4*-CKO) homozygous conditional knockout cell lines. Correct integration was confirmed by PCR (Table S1, primers 10 and 11 for Vtc1, and primers 19 and 20 for Vtc4).

### Western blot analyses

*T. cruzi* epimastigotes were harvested and washed twice with phosphate-buffered saline (pH 7.4). Parasites were resuspended in radioimmunoprecipitation assay buffer (150 mM NaCl, 20 mM Tris-HCl [pH 7.5], 1 mM EDTA, 1% SDS, and 0.1% Triton X-100) supplemented with a mammalian protease inhibitor cocktail (1:250), 1 mM phenylmethylsulfonyl fluoride, 2.5 mM tosyl phenylalanyl chloromethyl ketone, 100 µM *N*-(trans-epoxysuccinyl)-L-leucine 4-guanidinobutylamide (E64), and benzonase nuclease (25 U/mL of culture). Lysates were incubated on ice for 1 h, and protein concentrations were determined using a BCA protein assay. Equal amounts of protein (30 µg) from each sample were mixed with 4× Laemmli sample buffer (125 mM Tris-HCl [pH 7.0], 10% β-mercaptoethanol, 20% glycerol, 4% SDS, and 0.04% bromophenol blue), resolved on 10% SDS-polyacrylamide gels, and transferred onto nitrocellulose membranes using a Trans-Blot apparatus (Bio-Rad). Membranes were stained with Ponceau S to verify equal loading and transfer efficiency, followed by destaining with PBS containing 0.1% Tween 20 (PBS-T). Membranes were blocked overnight at 4°C in PBS-T containing 5% nonfat dry milk and then incubated for 1 h at room temperature with the following primary antibodies: mouse monoclonal anti-c-Myc (9E10; 1:1,000), mouse monoclonal anti-Ty1 (BB2; 1:2,500), mouse monoclonal anti-V5 (SV5-Pk1; 1:2,000), mouse monoclonal anti-tubulin (1:20,000), or rabbit polyclonal anti-TbVP1 (1:2,500). After three washes with PBS-T, membranes were incubated for 1 h at room temperature in the dark with IRDye 680RD-conjugated goat anti-rabbit IgG or IRDye 800CW-conjugated goat anti-mouse IgG secondary antibodies (1:10,000; LI-COR Biosciences). After three additional washes with PBS-T, blots were visualized using the Odyssey infrared imaging system (LI-COR Biosciences), and images were processed using Image Studio software. Alternatively, for chemiluminescent detection, membranes were incubated with horseradish peroxidase (HRP)-conjugated goat anti-mouse or goat anti-rabbit secondary antibodies (1:10,000), developed using Pierce ECL Western Blotting Substrate (Thermo Fisher Scientific), and imaged with a ChemiDoc Imaging System (Bio-Rad).

### Blue Native PAGE and immunodetection

*T. cruzi* epimastigotes (3 × 10⁸ cells) in the exponential growth phase were harvested by centrifugation at 1,000 × *g* for 10 min and washed twice with buffer A with glucose (BAG: 116 mM NaCl, 5.4 mM KCl, 0.8 mM MgSO_₄_, 50 mM HEPES, and 5.5 mM glucose; pH 7.3). Cells were resuspended in 1 mL of ice-cold lysis buffer (50 mM HEPES, pH 7.8; 50 mM NaCl; 0.3% Triton X-100; and cOmplete Mini EDTA-free Protease Inhibitor Cocktail) and incubated on ice for 30 min. Lysates were clarified by centrifugation at 21,000 × *g* for 15 min at 4°C, and the supernatant was transferred to a fresh tube. For molecular weight reference, 50 µg of bovine heart mitochondria (BHM; Abcam, ab110338) was solubilized in the same lysis buffer. Prior to loading, 1 µL of NativePAGE 5% G-250 sample additive (Thermo Fisher Scientific, BN2004) was added to each 25 µL sample. Samples were separated on a NativePAGE 4%–16% Bis-Tris protein gel. The gel strip containing BHM was excised and stained with Coomassie blue (0.3% Brilliant Blue R-250, 45% methanol, and 10% acetic acid), while the remaining gel containing parasite proteins was transferred to a polyvinylidene fluoride membrane. Membranes were probed with mouse monoclonal anti-c-Myc (9E10; 1:1,000), anti-Ty1 (BB2; 1:2,500), or anti-V5 (SV5-Pk1; 1:2,000) antibodies, washed three times with PBS containing 0.1% Tween 20, and incubated for 1 h at room temperature with HRP-conjugated goat anti-mouse or goat anti-rabbit secondary antibodies (1:10,000). Detection was performed using Pierce ECL Western Blotting Substrate (Thermo Fisher Scientific), and images were acquired with a ChemiDoc Imaging System (Bio-Rad).

### Immunofluorescence assays

*T. cruzi* epimastigotes were washed with PBS (pH 7.4) and fixed with 4% paraformaldehyde in PBS for 1 h at room temperature. Fixed cells were allowed to adhere to poly-L-lysine-coated coverslips and permeabilized for 5 min with 0.1% Triton X-100 in PBS. Cells were blocked overnight at 4°C in PBS containing 3% bovine serum albumin (BSA), 1% fish gelatin, 50 mM NH_₄_Cl, and 5% goat serum. Cells were incubated for 1 h at room temperature with primary antibodies diluted in PBS (pH 8.0) containing 1% BSA: mouse monoclonal anti-c-Myc (9E10; 1:100), mouse monoclonal anti-Ty1 (BB2; 1:100), mouse monoclonal anti-V5 (SV5-Pk1; 1:100), or rabbit polyclonal anti-TbVP1 (1:250). After three washes with PBS (pH 8.0) containing 1% BSA, cells were incubated for 1 h at room temperature in the dark with Alexa Fluor 488- or Alexa Fluor 546-conjugated goat anti-mouse or goat anti-rabbit secondary antibodies (1:1,000). Following labeling, cells were washed and mounted using Fluoromount-G containing 5 µg/mL DAPI. Differential interference contrast and fluorescence images were acquired under nonsaturating conditions using a 100× objective (numerical aperture 1.35) on an Olympus IX-71 inverted fluorescence microscope equipped with a Photometrics CoolSnap HQ CCD camera and controlled by DeltaVision software (Applied Precision, Issaquah, WA, USA). Images were processed with FIJI (ImageJ; National Institutes of Health, Bethesda, MD, USA).

### Southern blot analysis of *TcVtc1*-KO cells

Genomic DNA from WT and *TcVtc1*-KO epimastigotes was isolated by phenol-chloroform extraction, digested with PstI, and separated on a 0.8% agarose gel. DNA was transferred to a nylon membrane and hybridized with a biotin-labeled 375 nt fragment of *TcVtc1* (nt +124 to +498), amplified by PCR using WT genomic DNA as template (Table S1, primers 12 and 13). Probe labeling was performed with the Invitrogen BioPrime DNA Labeling System kit. Hybridization, post-hybridization washes, and detection were carried out using the Thermo Scientific Chemiluminescent Nucleic Acid Detection Module kit according to the manufacturer’s instructions. Signals were visualized using a ChemiDoc Imaging System (Bio-Rad).

### Southern blot analysis of *TcVtc4*-SKO cells

Genomic DNA from WT and *TcVtc4*-SKO epimastigotes was isolated by phenol-chloroform extraction, digested with EcoRV and BamHI, and separated on a 0.8% agarose gel. DNA was transferred to a nylon membrane and hybridized with a ^32^P-labeled 499 nt fragment of *TcVtc4* (nt +2471 to +2970), amplified by PCR using WT genomic DNA as template (Table S1, primers 26 and 27). Probe labeling was performed using [α-^32^P]dCTP (Perkin Elmer) with random hexanucleotide primers and the Klenow fragment of DNA polymerase (Prim-A-Gene Labeling System). Following hybridization and post-hybridization washes, detection was performed with a phosphor screen.

### Southern blot analysis of *TcVtc6*-KO cells

Genomic DNA from WT and *TcVtc6*-KO epimastigotes was isolated by phenol-chloroform extraction, digested with EcoRV and BamHI, and separated on a 0.8% agarose gel. DNA was transferred to a nylon membrane and hybridized with a biotin-labeled 411 nt fragment of TcVtc6 (nt +27 to +437), amplified by PCR using WT genomic DNA as template (Table S1, primers 36 and 37). Probe labeling was performed with the Invitrogen BioPrime DNA Labeling System kit. Hybridization, post-hybridization washes, and detection were carried out using the Thermo Scientific Chemiluminescent Nucleic Acid Detection Module kit according to the manufacturer’s instructions. Signals were visualized using a ChemiDoc Imaging System (Bio-Rad).

### *In vitro* metacyclogenesis

Metacyclogenesis was performed as described by Bourguignon et al. (57), with minor modifications. Epimastigotes were obtained after 4 days of growth in liver infusion tryptose medium and subjected to nutritional stress by incubation for 2 h at 28°C in triatomine artificial urine (TAU) medium containing 190 mM NaCl, 17 mM KCl, 2 mM MgCl_₂_, 2 mM CaCl_₂_, 0.035% sodium bicarbonate, and 8 mM phosphate (pH 6.9). Following the stress treatment, parasites were incubated for 96 h in TAU 3AAG medium, consisting of TAU medium supplemented with 10 mM L-proline, 50 mM sodium L-glutamate, 2 mM sodium L-aspartate, and 10 mM glucose. Cells released into the supernatant were collected and fixed in 4% paraformaldehyde prepared in PBS (pH 7.4) for 1 h at room temperature. Fixed parasites were allowed to adhere to poly-L-lysine-coated coverslips for 20 min at room temperature, washed with PBS (pH 7.4), and mounted onto glass slides using Fluoromount-G mounting medium containing 5 µg/mL 4’,6-diamidino-2-phenylindole (DAPI) for DNA staining. Metacyclic trypomastigotes were identified based on parasite morphology and the relative position of the nucleus and kinetoplast by fluorescence microscopy.

### Host cell invasion and intracellular replication

Gamma-irradiated (2,000 rad) Vero cells (4 × 10⁵ cells) were seeded onto sterile glass coverslips placed in 12-well plates and incubated overnight at 37°C in a humidified atmosphere containing 5% CO_₂_ in RPMI medium supplemented with 10% heat-inactivated fetal bovine serum. Tissue culture-derived trypomastigotes were incubated at 4°C overnight to allow amastigotes to settle, thereby separating them from motile trypomastigotes. Trypomastigotes collected from the supernatant were counted and used to infect Vero cells at a parasite-to-host cell ratio of 50:1. At 4 h post-infection, coverslips were washed extensively with D-Hanks’ balanced salt solution followed by phosphate-buffered saline (pH 7.4) to remove extracellular parasites. Cells were immediately fixed in 4% paraformaldehyde prepared in PBS (pH 7.4) for 30 min at 4°C. Coverslips were washed once with PBS and mounted onto glass slides using Fluoromount G containing 15 µg/mL 2-(4-aminophenyl)-1H-indole-6-carboxamidine (DAPI) to stain host and parasite DNA. Samples were examined using an Olympus BX60 fluorescence microscope. Infection was quantified by determining the percentage of host cells containing intracellular parasites and the number of intracellular parasites per infected cell in randomly selected microscopic fields. To assess amastigote replication, Vero cells were infected at a parasite-to-host cell ratio of 10:1 and incubated for 48 h at 37°C and 5% CO_₂_ prior to fixation and DAPI staining, as described above.

### Co-immunoprecipitation of TcVtc1-smV5/TcVtc4-smV5

*T. cruzi* epimastigotes (1 × 10⁸ cells) in the exponential growth phase were harvested by centrifugation at 1,000 × *g* for 10 min and washed twice with 5 mL of buffer A with glucose (116 mM NaCl, 5.4 mM KCl, 0.8 mM MgSO_₄_, 50 mM HEPES, and 5.5 mM glucose; pH 7.3) at room temperature. Cells were resuspended in 1 mL of ice-cold lysis buffer (50 mM HEPES, pH 7.8; 50 mM NaCl; 0.3% Triton X-100; and cOmplete Mini EDTA-free Protease Inhibitor Cocktail) and incubated on ice for 30 min. Lysates were cleared by centrifugation at 20,000 × *g* for 15 min at 4°C, and a portion of the supernatant was reserved for western blot analysis. The remaining soluble fraction was incubated with 20 µL of anti-V5 magnetic beads (Sigma, SAE0203) for 1 h at room temperature with gentle agitation to capture TcVtc1-smV5 or TcVtc4-smV5. Beads had been pre-washed twice with PBS (pH 7.4) and twice with lysis buffer using a magnetic rack. After incubation, the flow-through was collected, and the beads were washed three times with the lysis buffer, three times with 10 mM Tris-HCl and 50 mM NaCl (pH 7.4), and once with 50 mM NaCl. Proteins were eluted in 100 µL of elution buffer (50 µL 2× Laemmli sample buffer + 50 µL lysis buffer) and heated at 75°C for 10 min. Eluates were analyzed by western blot using an anti-V5 antibody.

### Analysis of TcVtc1 and TcVtc4 interactome

Co-immunoprecipitation of TcVtc1-smV5, TcVtc4-smV5, and WT epimastigote lysates was performed as described above using anti-V5 magnetic beads. After the final wash, beads were sent to the Proteomics & Metabolomics Facility at the Nebraska Center for Biotechnology (University of Nebraska-Lincoln) for mass spectrometry analysis, where the samples were heated in 70 µL reducing sample buffer at 95°C for 10 min prior to loading 50 µL of sample onto a Bolt 12% Bis-Tris-Plus gel (Thermo Fisher Scientific). The gel was fixed for 1 h, washed with water briefly, and stained with colloidal Coomassie blue G250 stain overnight, then destained before excising the whole lane. The gel slices were cut up and washed with ammonium bicarbonate/acetonitrile to remove SDS and CBB stain, reduced with DTT, and alkylated with iodoacetamide. Trypsin was added, and digestion was carried out overnight at 37°C. Peptides were extracted from the gel pieces and dried down in a Speed-Vac. The digests were redissolved in 20 µL of 5% acetonitrile and 0.05% trifluoroacetic acid, and 5 µL was injected. Analysis was carried out as previously described (58) using a 2 h gradient on a 0.075 mm × 250 mm C18 Waters CSH column at 45°C using an RSLCnano LC system (Thermo Fisher Scientific) coupled to an Orbitrap Eclipse mass spectrometer (Thermo Fisher Scientific). The mobile phases were made of 0.1% formic acid in water (A) and 0.1% formic acid and 100% acetonitrile (B) and were run using a linear gradient at 300 nL/min at 4% (*t* = 0–3 min), then to 22% B (*t* = 105 min), to 80% B (*t* = 107 min), holding at 80% B (*t* = 109.8 min) before returning to 4% B (*t* = 110 min). The spray voltage in the ion source was set at 2,000 V while operating in static gas mode (gas off). The mass spectrometer was run in data-dependent mode using a mass range of mass/charge ratio (*m/z*) 375–1,500, a 50 ms injection time, and AGC target of 4e^5^ for the MS1. Data-dependent MS2 spectra were acquired in the Orbitrap detector using HCD fragmentation at a normalized collision energy of 30%, a resolution of 15,000, a dynamic exclusion time of 45 s, a quadrupole isolation window of 0.7 *m/z*, and a total cycle time of 2 s. All MS/MS samples were analyzed using Mascot (Matrix Science, London, UK; version 2.7.0). Mascot was set up to search the cRAP_20150130.fasta; TriTrypDB-68_TcruziYC6_AnnotatedProteins_20241208 database (14,268 entries), assuming the digestion enzyme trypsin. Mascot was searched with a fragment ion mass tolerance of 0.060 Da and a parent ion tolerance of 10.0 PPM. Deamidation of asparagine and glutamine, oxidation of methionine, and carbamidomethylation of cysteine were specified in Mascot as variable modifications. Scaffold (version 5.3.3) was used with a peptide probability > 80% (Peptide Prophet) and protein probability > 99% with at least two peptides (Protein Prophet). Proteins containing similar peptides were grouped to satisfy parsimony principles. Proteins were considered enriched in TcVtc1-smV5 or TcVtc4-smV5 samples if their abundance showed a fold change > 2 relative to the WT control. Shared proteins were defined as those enriched in both data sets. Gene ontology enrichment analysis of shared proteins was performed using TriTrypDB, with a significance threshold of *P* < 0.06. As this experiment included only a single replicate per condition, the analysis was considered exploratory, and no statistical tests were applied to fold-change values.

### Reciprocal co-immunoprecipitation of TcVtc4-Ty1/TcVtc6-3×c-Myc cell line

*T. cruzi* epimastigotes (1 × 10⁸ cells) in the exponential growth phase were harvested by centrifugation at 1,000 × *g* for 10 min and washed twice with 5 mL of buffer A with glucose at room temperature. Cells were resuspended in 1 mL of ice-cold lysis buffer (50 mM HEPES, pH 7.8; 50 mM NaCl; 0.3% Triton X-100; and cOmplete Mini EDTA-free Protease Inhibitor Cocktail) and incubated on ice for 30 min. Lysates were cleared by centrifugation at 20,000 × *g* for 15 min at 4°C, and a portion of the supernatant was reserved for western blot analysis. The remaining soluble fraction was incubated with 50 µL of anti-Ty1 magnetic beads and 25 µL of anti-c-Myc magnetic beads for 1 h at room temperature with gentle agitation to capture TcVtc4-Ty1 and TcVtc6-3×c-Myc, respectively. Beads had been pre-washed twice with PBS (pH 7.4) and twice with lysis buffer using a magnetic rack. After incubation, the flow-through was collected, and the beads were washed three times with the lysis buffer, three times with 10 mM Tris-HCl and 50 mM NaCl (pH 7.4), and once with 50 mM NaCl. Proteins were eluted in 60 µL of elution buffer (15 µL of 4× Laemmli sample buffer + 45 µL lysis buffer) and heated at 75°C for 10 min. Eluates were analyzed by western blot. To assess reciprocal co-immunoprecipitation, membranes were incubated with anti-Ty1 and anti-c-Myc antibodies to detect the presence of TcVtc4-Ty1 and TcVtc6-3×c-Myc in both pull-downs.

### Short-chain polyP extraction using titanium dioxide beads

*T. cruzi* epimastigotes (1 × 10⁹ cells) were harvested and washed twice with BAG. The cell pellet was resuspended in 1 M perchloric acid, sonicated at 40% amplitude for 10 s, and incubated on ice for 15 min. Samples were clarified by centrifugation at 18,000 × *g* for 5 min, and the supernatant was transferred to fresh tubes. Titanium dioxide beads (4 mg; Titansphere TiO_₂_, 5 μm, GL Sciences) were washed with water, followed by 1 M perchloric acid, and then added to the supernatant. The mixture was rotated for 30 min at 4°C. Beads were collected by centrifugation at 3,500 × *g* for 1 min at 4°C, and bound short-chain polyP were eluted with 2.8% ammonium hydroxide. Eluates were neutralized with perchloric acid, mixed with Orange G loading buffer, and resolved by PAGE using a 35% acrylamide/bisacrylamide (19:1) gel in Tris/Borate/EDTA buffer, as described by Losito et al. (59). Gels were stained in toluidine blue solution (0.05% toluidine blue, 20% methanol, and 2% glycerol) for 30 min at room temperature and destained for 2 h with multiple changes of the same solution without dye. Images were acquired on a white-light transilluminator, and densitometric analyses were performed using ImageJ software.

### Statistical analysis

Statistical analyses were performed using GraphPad Prism software (version 10; GraphPad Software, La Jolla, CA, USA). Data are presented as mean ± standard deviation or standard error of the mean, as indicated in the figure legends, from *n* independent biological experiments. Statistical significance was assessed using an unpaired two-tailed Student’s *t* test for comparisons between two groups, or one-way analysis of variance for comparisons among more than two groups.

## SUPPLEMENTAL MATERIAL

Supplemental figures mbio.00372-26-s0001.pdf Figures S1 to S5.

Table S1 mbio.00372-26-s0002.xlsx Oligonucleotides used in this work.

Table S2 mbio.00372-26-s0003.xlsx Mass spectrometry analysis.

ASM does not own the copyrights to Supplemental Material that may be linked to, or accessed through, an article. The authors have granted ASM a non-exclusive, world-wide license to publish the Supplemental Material files. Please contact the corresponding author directly for reuse.
