# Supplementary material for: Composition and role of the vacuolar transporter chaperone complex in polyphosphate synthesis and infectivity in Trypanosoma cruzi
Source: mBio. 2026 May 13;17(6):e00372-26. doi: 10.1128/mbio.00372-26 (PMC13251457; doi:10.1128/mbio.00372-26)
Supplement: Supplemental figures — Figures S1 to S5. [file mbio.00372-26-s0001.pdf]

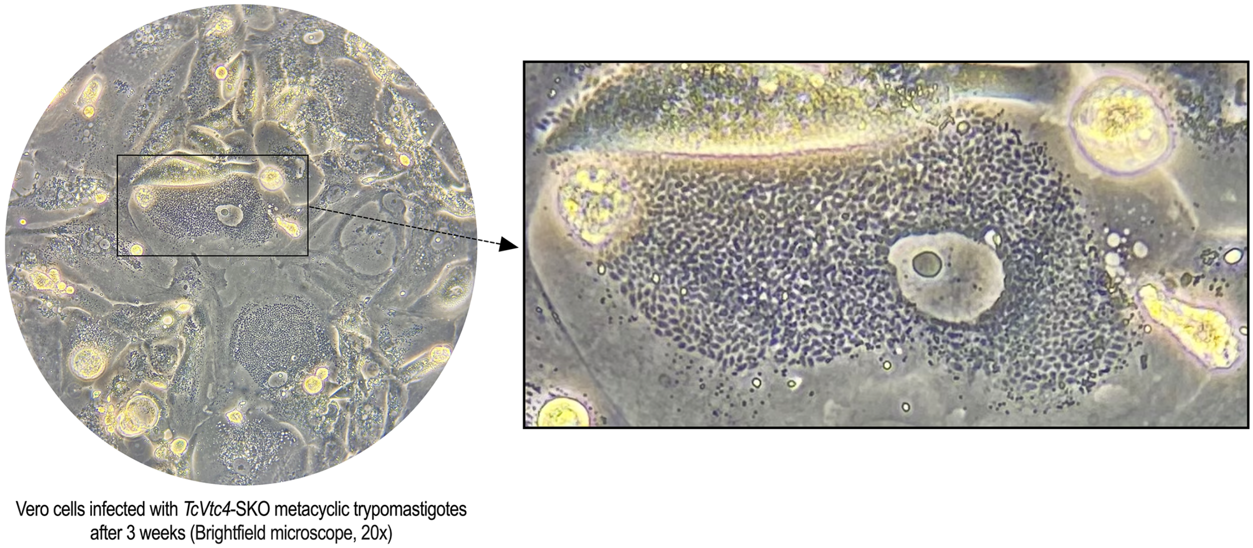

Figure S1. Infection of Vero cells with *TcVtc4*-SKO metacyclic trypomastigotes. Vero cells were incubated with *TcVtc4*-SKO metacyclic trypomastigotes. After 3 weeks, the mammalian cells were full of amastigotes, but they could not differentiate into trypomastigotes to continue the life cycle. Brightfield microscope, 20x.

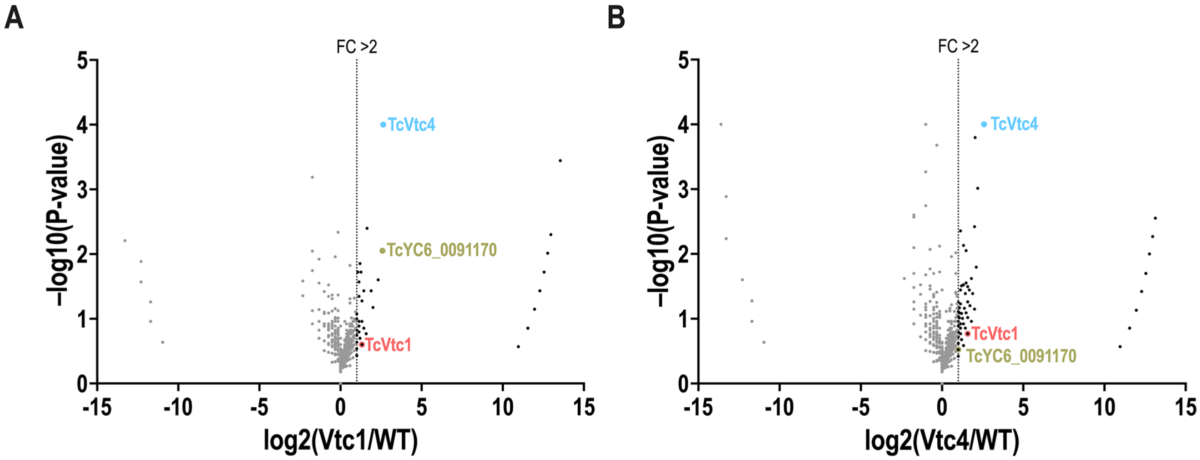

Figure S2. Volcano plots of proteins enriched in TcVtc1-smV5 and TcVtc4-smV5 co-immunoprecipitations. (A) Volcano plot showing proteins enriched in TcVtc1-smV5 samples relative to wild-type control. Gray dots indicate proteins with fold change ≤2, and black dots indicate proteins with fold change >2. Vertical dashed lines indicate the fold change threshold. (B) Volcano plot showing proteins enriched in TcVtc4-smV5 samples relative to wild-type control. Gray dots indicate proteins with fold change ≤2, and black dots indicate proteins with fold change >2. Vertical dashed lines indicate the fold change threshold.

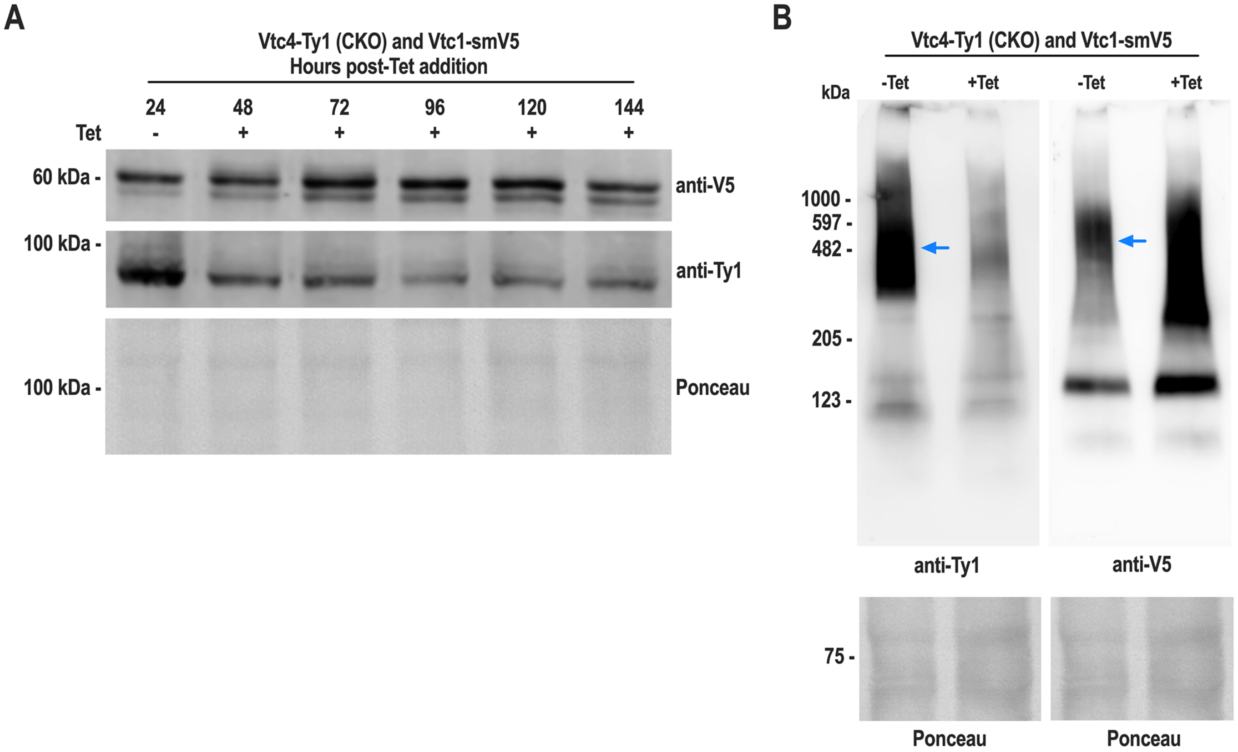

Figure S3. Analysis of Vtc1 interaction with Vtc4 in the TcVtc4-Ty1 conditional knockdown cell line. (A) Western blot analysis of TcVtc4-Ty1 and TcVtc1-smV5 expression over time in the TcVtc4-Ty1/TcVtc1-smV5 cell line treated with tetracycline. Samples were collected at 24, 48, 72, 96, 120, and 144 hours post-treatment and probed with anti-Ty1 and anti-V5 antibodies. Ponceau was used as a loading control. (B) Blue Native PAGE analysis of TcVtc1-smV5 and TcVtc4-Ty1 protein complexes in the TcVtc4-Ty1/TcVtc1-smV5 cell line in the presence or absence of tetracycline. Lysates were separated under native conditions and analyzed by western blot using anti-V5 and anti-Ty1 antibodies. Ponceau was used as a loading control. Blue arrows indicate the bands corresponding to the potential complexes.

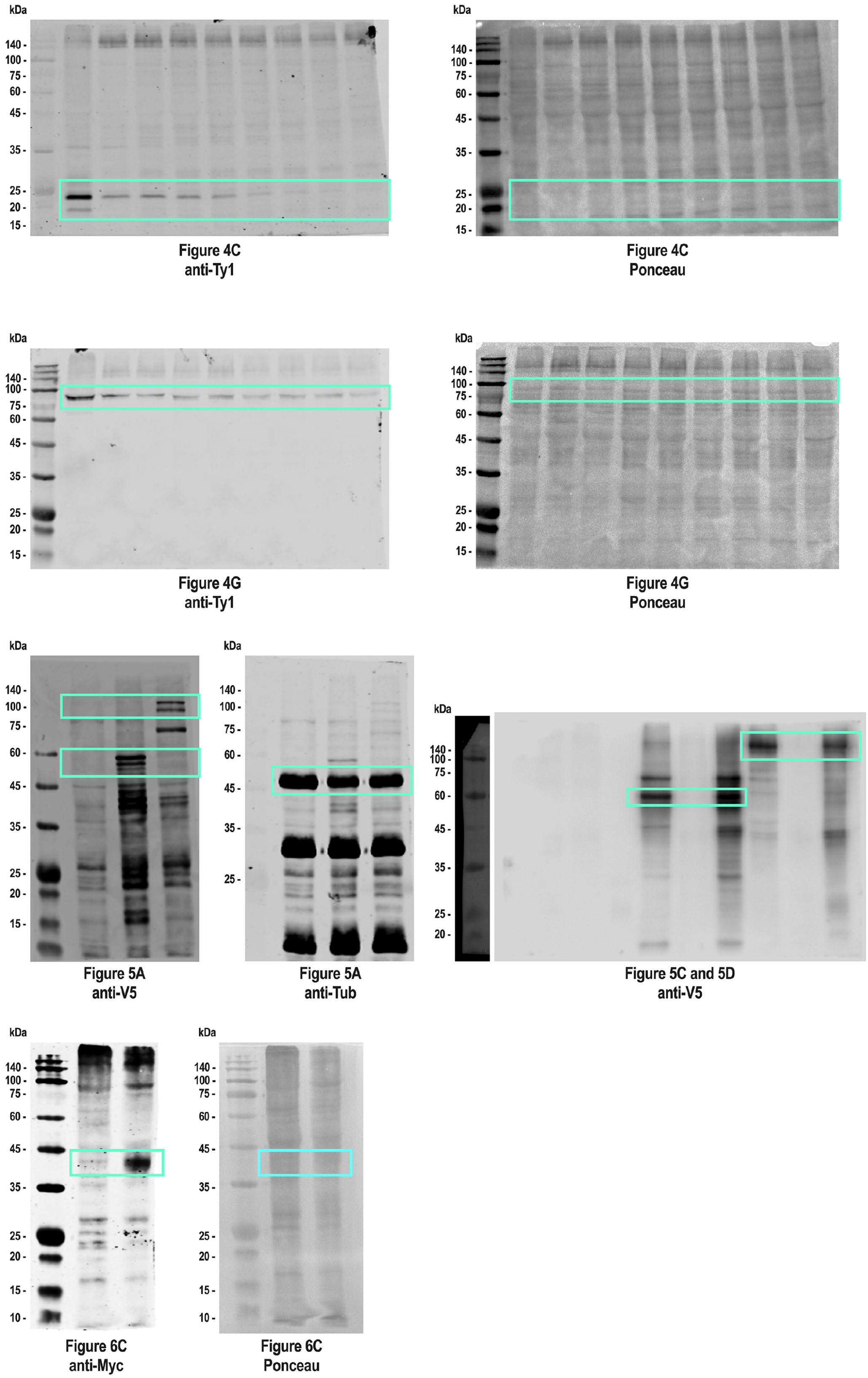

Figure S4. Complete western blots shown in this work.

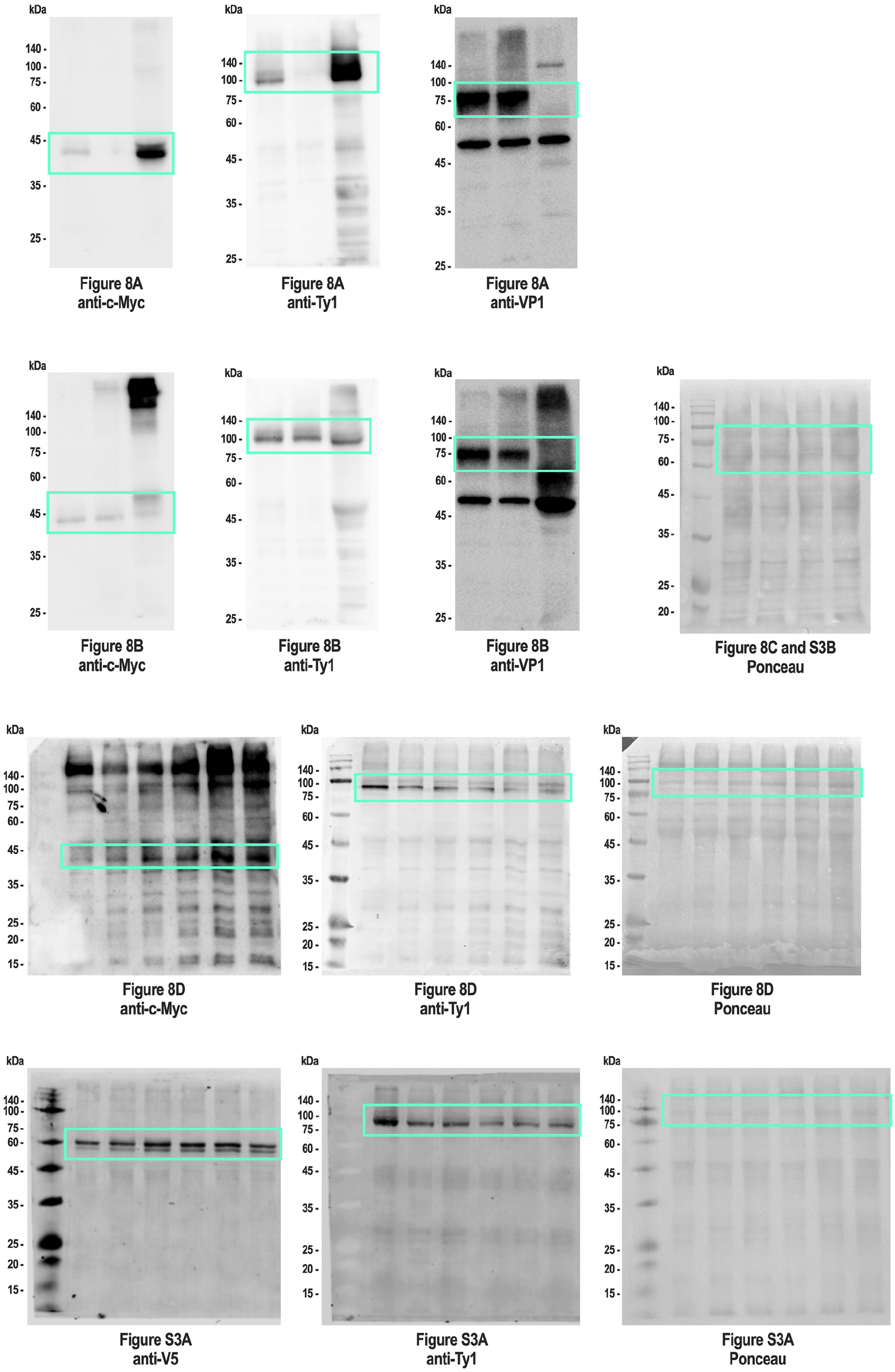

Figure S5. Complete western blots shown in this work
